# Discovery of key molecular signatures for diagnosis and therapies of glioblastoma by combining supervised and unsupervised learning approaches

**DOI:** 10.1038/s41598-024-79391-2

**Published:** 2024-11-11

**Authors:** Arnob Sarker, Md. Abdul Aziz, Md. Bayazid Hossen, Md. Manir Hossain Mollah, Al-Amin, Md. Nurul Haque Mollah

**Affiliations:** 1 Department of Biochemistry and Molecular Biology, University of Rajshahi, Rajshahi, 6205 Bangladesh; 2 Bioinformatics Lab (Dry), Department of Statistics, University of Rajshahi, Rajshahi, 6205 Bangladesh; 3 Department of Agricultural and Applied Statistics, Bangladesh Agricultural University, Mymensingh, 2202 Bangladesh; 4 Department of Physical Sciences, Independent University, Bangladesh (IUB), Dhaka, Bangladesh; 5 Department of Zoology, University of Rajshahi, Rajshahi, 6205 Bangladesh

**Keywords:** Glioblastoma, Gene expression profiles, Key genes, Drug repurposing, Bioinformatics and machine learning approaches, Cancer, Computational biology and bioinformatics, Drug discovery, Systems biology

## Abstract

Glioblastoma (GBM) is the most malignant brain cancer and one of the leading causes of cancer-related death globally. So, identifying potential molecular signatures and associated drug molecules are crucial for diagnosis and therapies of GBM. This study suggested GBM-causing ten key genes (ASPM, CCNB2, CDK1, AURKA, TOP2A, CHEK1, CDCA8, SMC4, MCM10, and RAD51AP1) from nine transcriptomics datasets by combining supervised and unsupervised learning results. Differential expression patterns of key genes (KGs) between GBM and control samples were verified by different independent databases. Gene regulatory network (GRN) detected some important transcriptional and post-transcriptional regulators for KGs. The KGs-set enrichment analysis unveiled some crucial GBM-causing molecular functions, biological processes, cellular components, and pathways. The DNA methylation analysis detected some hypo-methylated CpG sites that might stimulate the GBM development. From the immune infiltration analysis, we found that almost all KGs are associated with different immune cell infiltration levels. Finally, we recommended KGs-guided four repurposable drug molecules (Fluoxetine, Vatalanib, TGX221 and RO3306) against GBM through molecular docking, drug likeness, ADMET analyses and molecular dynamics simulation studies. Thus, the discoveries of this study could serve as valuable resources for wet-lab experiments in order to take a proper treatment plan against GBM.

**Supplementary Information:**

The online version contains supplementary material available at 10.1038/s41598-024-79391-2.

## Introduction

Glioblastoma (GBM) is the grade-IV brain tumor in Gliomas according to the clinical and histopathological characteristics. Several genetic disorders like loss of heterozygosity, amplification, deletion, and mutation are associated with the initiation and development of GBM. Also, DNA methylation at CpG site in the promoter region of a gene is considered as a major cause of GBM. It affects the brain and central nervous system, accounting for approximately 14.3% of all tumors and comprising 49.1% of all malignant tumors^1–3^. Patients with GBM have only 3–5% survival rate for more than 5 years^4^. Despite the recent advancements in multimodality therapy like chemotherapy, radiotherapy, and supportive care, the overall prognosis for GBM patients remains unsatisfactory, and recurrence of the disease is frequently observed^5,6^. So, discovering new potential molecular biomarkers might play a crucial role in advancing GBM diagnosis, prognosis and therapies^7^. Because of the rapid expansion of high-throughput platforms, the vast amount of microarray gene expression data is generating rapidly associated with different diseases. Bioinformatics approaches are playing the significant role in identifying potential genomic biomarkers from those gene expression profiles for promptly diagnosis and therapies of diseases^8–11^.

**Fig. 1:**
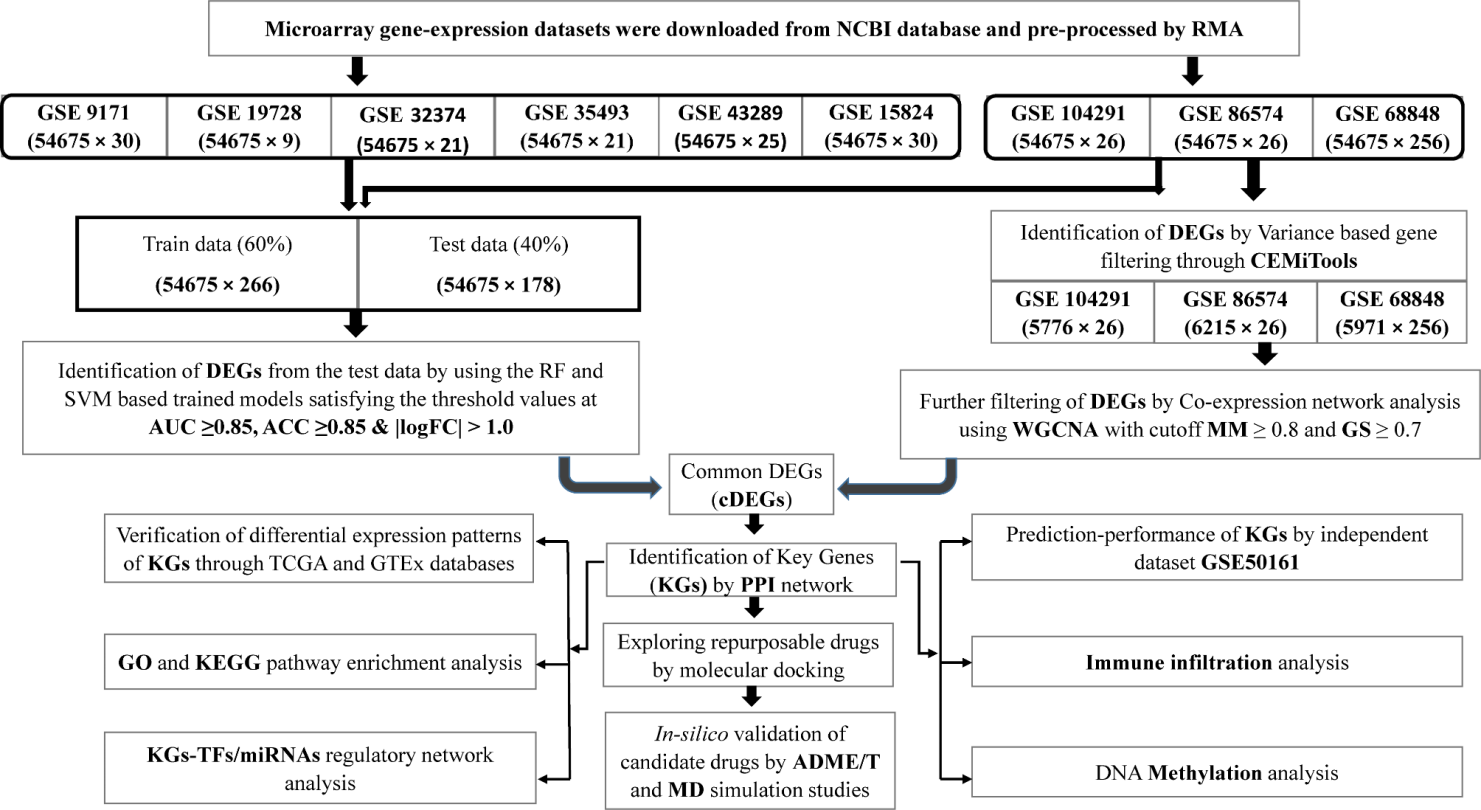
The workflow of the study.

There are some bioinformatics studies that have explored GBM-causing key genes (KGs) highlighting their pathogenetic processes through unsupervised WGCNA^11–14^ and LIMMA^15–25^ approaches. However, we observed that their KGs-sets are not so consistent and none of these studies provided KGs-guided therapeutic indications against GBM, though a therapeutic drug kills cancer cells by targeting the cancer-causing genes/proteins^26,27^. In this study, we attempted to explore more consistent GBM-causing KGs by analyzing multiple gene expression profile datasets generated from different countries using both supervised and unsupervised learning approaches for diagnostic and therapeutic indications,

since supervised learning compared to the unsupervised learning gives more accurate results^28,29^. The workflow of this study is given in Fig. 1.

## Materials and methods

### Source and description of data

In order to achieve our objectives, we considered both the raw data and metadata related to GBM as introduced below:

#### Collection of gene expression profiles from online databases

Total 10 microarray gene-expression profile datasets that contained GBM and control samples, were downloaded from the National Center for Biotechnology Information (NCBI) Gene Expression Omnibus (GEO) database to explore GBM-causing key genes. The detail description of the datasets was given in Table 1.

**Table 1:** Information of gene-expression profile datasets associated with GBM.

Accession ID for the Datasets	Country		GBM	Control
GSE104291	Switzerland		24	2
GSE86574	USA		15	11
GSE68848	USA		228	28
GSE15824	Switzerland		25	5
GSE9171	USA		30	0
GSE19728	China		5	4
GSE32374	USA		21	0
GSE35493	USA		12	9
GSE43289	Spain		25	0
GSE50161	USA		34	13

#### Collection of meta-drug molecules from online sources

In order to repurpose potential drug molecules, we collected in total 139 drug molecules associated with GBM-causing genes from online databases DSigDB^30^ and GSCALite^31^, and published articles^32,33,42,34–41^ (Table S1).

### Identification of differentially expressed genes (DEGs) by unsupervised approaches

To explore differentially expressed genes (DEGs) between GBM and control samples by the unsupervised approaches, we considered three microarray gene expression profile datasets (GSE104291, GSE86574 and GSE68848) from Table 1. To remove unimportant genes from each of these three datasets, at first, we considered variance-based gene filtering by CEMiTool^43^. For testing the significance of *i*th gene, this tool computes *p*-value as follows

1 $$\:{p}_{i}=\text{P}\text{r}[{\sigma\:}^{2}\ge\:\:{s}_{i}^{2}],$$

where $\:{\sigma\:}^{2}$ follows inverse gamma distribution and $\:{s}_{i}^{2}$ is the variance for the expressions of *i*th gene. Obviously, $\:{p}_{i}=1$ for $\:{s}_{i}^{2}=0,$ while $\:{p}_{i}=0$ for $\:{s}_{i}^{2}=\infty\:,$ which indicates *p*-value decreases due to the increasing of $\:{s}_{i}^{2}.$ Now $\:{s}_{i}^{2}=0$ indicates all expressions in both case and control groups are equal. That is, *i*th gene is EE (equally expressed) between case and control group. It may be mentioned here that a gene is said to be equally expressed (EE) if its average expressions in case and control groups are equal; otehrwise, it is said to be differentially expressed (DE). It can also be shown that variance of differentially expression patterns is greater than variance of equally expression patterns for *i*th gene, that is $\:{s}_{i,\:\:DE}^{2}>{s}_{i,\:\:EE}^{2}$ (see supplimentary section S1). Therefore, *p*-values computed by Eq. (1) can be used to select the differentially expressed genes (DEGs). This study considered *p*-values < 0.05 to select the DEGs. Then these DEGs sets were further filtered by using weighted correlation network analysis (WGCNA)^44^, which finds clusters (modules) of highly correlated genes. The WGCNA r-package was used to construct the co-expression network and gene modules for each of the three datasets. Module-trait relationships were determined by calculating the Pearson correlation coefficient between module eigengenes (MEs) and traits. Modules with significant correlations (>|0.6|, p-value < 0.005) were selected for further analysis. Further, signature genes were selected with module membership (MM) ≥ 0.8 and gene significance (GS) ≥0.7 as the cutoff value, also considered as DEGs for this study (Table S2).

### Identification of DEGs) by supervised approaches

Random Forest (RF)^45^ and Support Vector Machine (SVM)^46^ are both popular supervised machine learning techniques for sample classifications. To explore differentially expressed genes (DEGs) between GBM and control samples by these two supervised approaches, we considered additional six datasets (GSE15824, GSE9171, GSE19728, GSE32374, GSE35493, GSE43289) from Table 1 with the previous three datasets (GSE104291, GSE86574 and GSE68848) that were analyzed by the unsupervised approaches as displayed in Fig. 1. It should be noted here that supervised approach requires more samples than unsupervised approaches to identify DEGs, since supervised approach requires more samples for partitioning dataset into training and test sets. We implemented both RF and SVM models to identify DEGs between GBM and control samples by the following steps.

#### Step 1

We combined nine preprocessing datasets from Table 1 to create a larger dataset of 444 with case and control.

#### Step 2

Then, for the *i*th gene (*i* = 1, 2, …, N), we trained both the RF and SVM prediction models by using randomly selected 60% of the total samples and leaving the remaining 40% as the test dataset. We implemented R-packages “randomForest” and “e1071” to train RF and SVM models, respectively.

#### Step 3

The trained model was used to classify the remaining 40% samples with the *i*th gene (*i* = 1, 2, …, N).

#### Step 4

After that, for the *i*th gene (*i* = 1, 2, 3,…,N), we computed the area under the ROC curve (AUC) and the classification accuracy (ACC) at a false positive rate (FPR) of 0.10 for both RF and SVM prediction models with the test samples.

#### Step 5

Finally, we detected the up- and down-regulated DEGs by satisfying the following criterion.

(i) Up-regulated DEGs if AUC_i_ ≥0.85, ACC_i_ ≥0.85 for both prediction models & logFC_i_ > 1.
(ii) Down-regulated DEGs if AUC_i_ ≥0.85, ACC_i_ ≥0.85 for both prediction models & logFC_i_ < -1.

Where logFC = log [(mean of controls) / (mean of cases)] indicates the log of fold change (logFC) value.

### Selection of common DEGs (cDEGs) detected by both supervised and unsupervised approaches

From two DEGs lists computed by supervised and unsupervised methods respectively, we considered their common DEGs (cDEGs) as the most potential GBM-causing genes (cDEGs). Subsequently, these cDEGs were visualized by Venn diagram.

### Protein-protein interaction (PPI) network analysis of cDEGs

To explore key genes (KGs), an online database and analysis tool (STRING v11.5) was used to create the PPI network of cDEGs. The network was visualized using the Cytoscape software^47^. The CytoHubba^48^ plugin in Cytoscape was used to select KGs based on six different topological measures including Closeness, Degree, Maximum Neighborhood Component (MNC), Edge Percolated Component (EPC), Maximal Clique Centrality (MCC) and Density of Maximum Neighborhood Component (DMNC). Further the “Molecular Complex Detection” (MCODE) plugin in Cytoscape was employed to detect the most prominent modules within the PPI network^49^.

### In-silico verification of KGs using independent expression profiles

To verify the differential expression patterns of KGs, we used TCGA and GTEx databases from the GEPIA2 web tool^50^. We constructed Box plots to confirm the differential expression patterns of KGs between GBM and control groups. Also, to evaluate the predictive ability of KGs, we constructed prediction model based on Random Forest (RF) using independent expression profiles collected from NCBI database with accession ID GSE50161 (Table 1) and draw the ROC curves using the R-package “ROCR”^51^ to evaluate the prediction performance.

### Enrichment analysis of KGs with GO terms and KEGG pathways

The Gene Ontology (GO) project is a bioinformatics tool that uses domain-specific ontologies to provide a complete source of functional data on gene products and descriptions of activities^52^. To investigate the Gene Ontology and KEGG pathway of KGs, we considered GeneCodis^53^, David^54^ and Enrichr^55^ database with P-value of 0.05 was chosen as threshold.

### KGs regulatory network analysis

We investigated how transcription factors (TFs) and microRNAs (miRNAs) regulate KGs at both the transcriptional and post-transcriptional stages by analyzing their regulatory networks. The JASPAR database^56^ was used to identify the main TFs and the TarBase database^57^ was used to explore the main miRNAs. The NetworkAnalyst serve r^58^ was used to produce the networks. We used Cytoscape to visualize their interaction networks^47^.

### DNA methylation analysis of KGs

MethSurv^59^ and ULCAN^60^ was used to investigate the methylation status of the KGs in GBM. Both of these web servers utilize TCGA methylation data. The level of DNA methylation was expressed by β -values (with a range from 0 to 1). M / (M + U + 100) is the formula used to calculate the β -values. The methylated and unmethylated intensities are denoted by M and U, respectively.

### Immune infiltration level analysis of KGs

The Tumor Immune Estimation Resource (TIMER 2.0)^61^ is a comprehensive tool that estimates the quantity of tumor-infiltrating immune cell types from TCGA data. We utilized TIMER’s online tools to investigate the immune infiltration levels of CD8 + T cells, CD4 + T cells, neutrophils, B cells, macrophages, and dendritic cells with KGs in GBM.

### Drug repurposing

To explore repurposable drug molecules, we performed molecular docking, Drug-Likeness and ADMET analysis, and MD simulation studies as discussed below.

#### Molecular docking

We considered 10 kg and associated top 2 TFs proteins as the target receptors. To explore potential ligands or drug molecules for treating GBM, molecular docking analysis between receptors and ligands was performed. Receptor proteins’ 3D structures were obtained from SWISS-MODEL^62^, Protein Data Bank^63^, and AlphaFold databases^64^. All 139 GBM-related meta-drug candidates’ 3D structures were taken from the PubChem database^65^. Following this, the binding affinity scores (in kcal/mol) between receptors and ligands (drug molecules) were determined through molecular docking using AutoDock Vina^66^. The arrangement of receptor proteins was based on the descending order of the average values in each row and drug-agents were arranged by the decreasing-order of column average in the score matrix to choose the top-ranked candidate drug molecules.

#### Evaluation of drug-likeness and ADMET properties of top-ranked drugs

We explored the structural features and chemical descriptors of the top ranked 25 drug molecules to understand their drug-like properties and assess their ADMET characteristics. We utilized SCFBio web tool to assess whether the compounds satisfied the Lipinski rule criteria^67^. Then, ADMET properties were computed by SwissADME^68^, amdetSAR^69^ and pkCSM^70^ for predicting the AMDET parameters. Further the interactions between these drugs and the top receptor protein were analyzed by PyMol^71^ and the Protein–Ligand Interaction Profiler (PLIP) web service^72^ by analyzing the docked complexes.

#### Molecular dynamic (MD) simulations studies

We carried out MD simulations by YASARA software^73^ and the AMBER14^74^ force field to investigate the dynamic properties of the top protein-ligand complexes. The hydrogen bonding network of the selected complexes was optimized and submerged using the TIP3P water model before the simulation was performed^75^. To maintain periodic boundary conditions, the solvent density was adjusted to 0.997 g/ml. Each simulation underwent a preliminary energy minimization process using steepest gradient algorithm with 5000 cycles. Each simulation was conducted under typical physiological conditions (298 K temperature, pH 7.4, 0.9% NaCl)^76^ and employed a multiple time-step algorithm^77^ which involved 2.50 femtoseconds (fs) time-step interval. A 100 ns molecular dynamics simulation was performed with a Berendsen thermostat^78^, and constant pressure. These conditions helped create a stable and realistic environment for the simulation. Trajectories of the simulation were captured at regular intervals of 250 picoseconds (ps), providing snapshots of the system’s behavior for subsequent in-depth analysis. The YASARA^79^ macro’s default script and the SciDAVis were used to conduct the primary analysis. Following that, using the YASARA software, all snapshots were accounted for MM-PBSA (MM-Poisson-Boltzmann surface area) binding free energy calculations. The following formula is used to calculate the binding free energy of the MM-PBSA^80^:

$$\begin{aligned} {\text{Binding free Energy}}\,= & \,{{\text{E}}_{{\text{potReceptor}}}}+{\text{ }}{{\text{E}}_{{\text{solvReceptor}}}}+{\text{ }}{{\text{E}}_{{\text{solvLigand}}}} \\& +{\text{ }}{{\text{E}}_{{\text{potLigand}}}} - {{\text{E}}_{{\text{solvComplex}}}} - {{\text{E}}_{{\text{potComplex}}}} \\ \end{aligned}$$

It is important to note that larger positive energies in the results indicated more favorable and stronger binding^81^.

## Results

### Identification of DEGs by unsupervised approaches

**Fig. 2:**
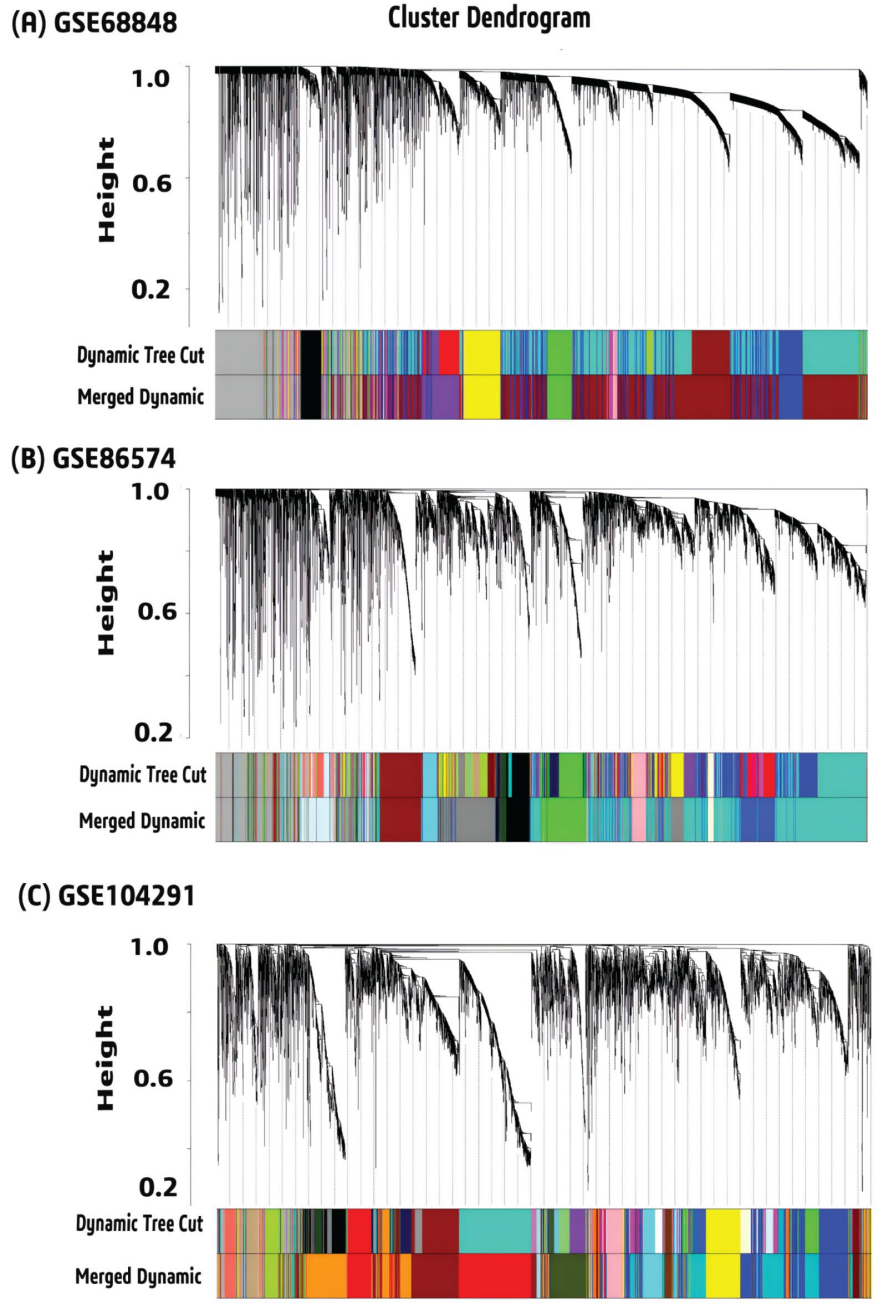
Cluster dendrogram of merged and unmerged modules for **(A)** GSE68848 **(B)** GSE86574 and **(C)** GSE104292.

At first, we detected 5971, 6215 and 5776 DEGs from GSE68848, GSE86574 and GSE104292 respectively based on the variance property of genes expression with “CEMiTool”. For each filtered gene expression matrices, a soft threshold β value was chosen (11 for GSE68848, 18 for GSE86574 and 15 for GSE104291) based on a cutoff R^2^ value of 0.8 (Figure S1). Following that, some modules were found by hierarchical clustering with the minimal module size 30. To merge the modules, cut height of module eigengene was set to 0.1 for GSE68848, 0.15 for GSE86574 and, 0.30 for GSE104291 (Figure S2). All gene co-expression modules before and after merging were visualized in (Fig. 2). To uncover the relationship between modules and clinical traits (GBM and control), we selected six modules from GSE68848, six modules from GSE86574 and four modules from GSE104291 based on the module-trait relationship (>|0.6|, p-value < 0.005) (Figure S3). Further, a total of 699 signature genes (DEGs) were found from those significant modules with the cutoff at MM ≥ 0.8 and GS ≥ 0.7 (Figure S4). Combinedly, we got 502 DEGs between GBM and control samples (Table S4).

### Identification of differential expressed genes (DEGs) by machine learning (ML) approaches

**Fig. 3:**
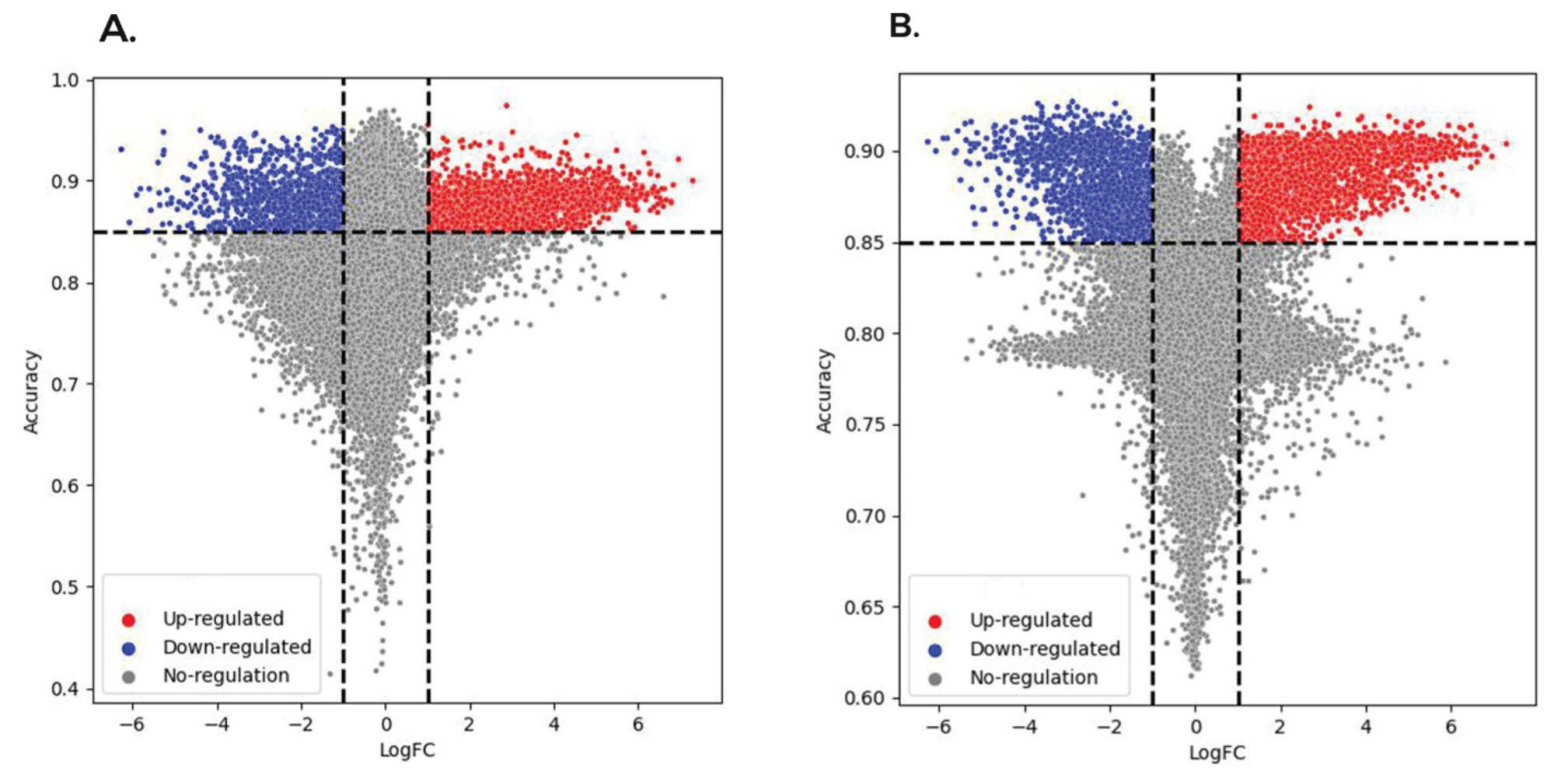
The volcano plot based on ACC and LogFC. **(A)** DEGs selection by RF-based prediction model **(B)** DEGs selection by SVM-based prediction model.

We calculated AUC and ACC values for each gene in order to detect DEGs by using RF and SVM based prediction model as described in Sect. 2.2. Then we separated upregulated and down regulated DEGs by using logFC values. We obtained 1123 common DEGs, where 742 are downregulated, 381 upregulated, by using the criterion as given in step 5 of Sect. 2.2 (Fig. 3 and Table S3).

### Selection of common DEGs (cDEGs) for ML and WGCNA approaches

**Fig. 4:**
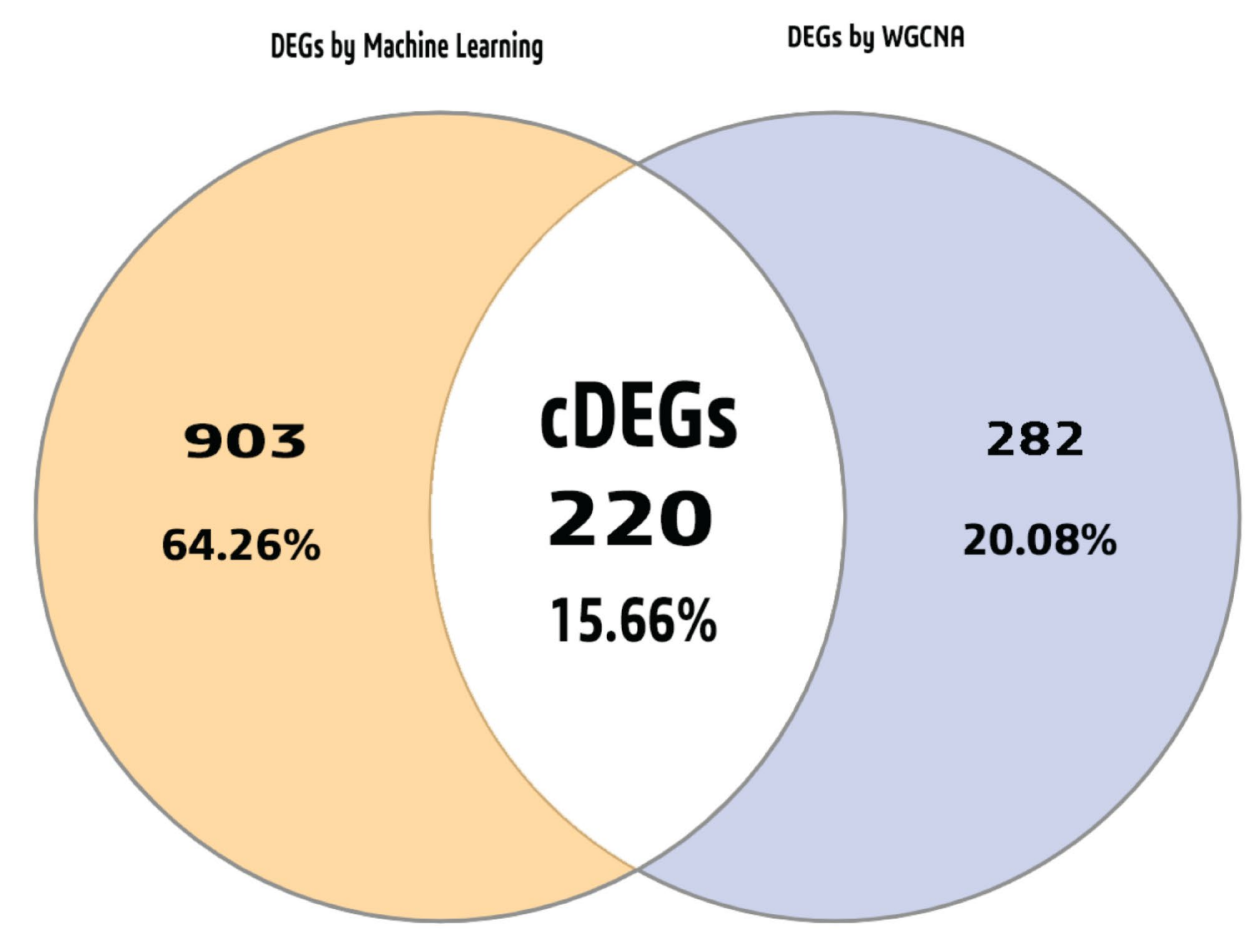
The cDEGs between the ML and WGCNA techniques are depicted in a Venn diagram.

Total 220 cDEGs were identified as GBM causing genes. These cDEGs were visualized using a Venn diagram in Fig. 4 (see also Table S5).

### Key gene (KG) identification from cDEGs by PPI network analysis

**Fig. 5:**
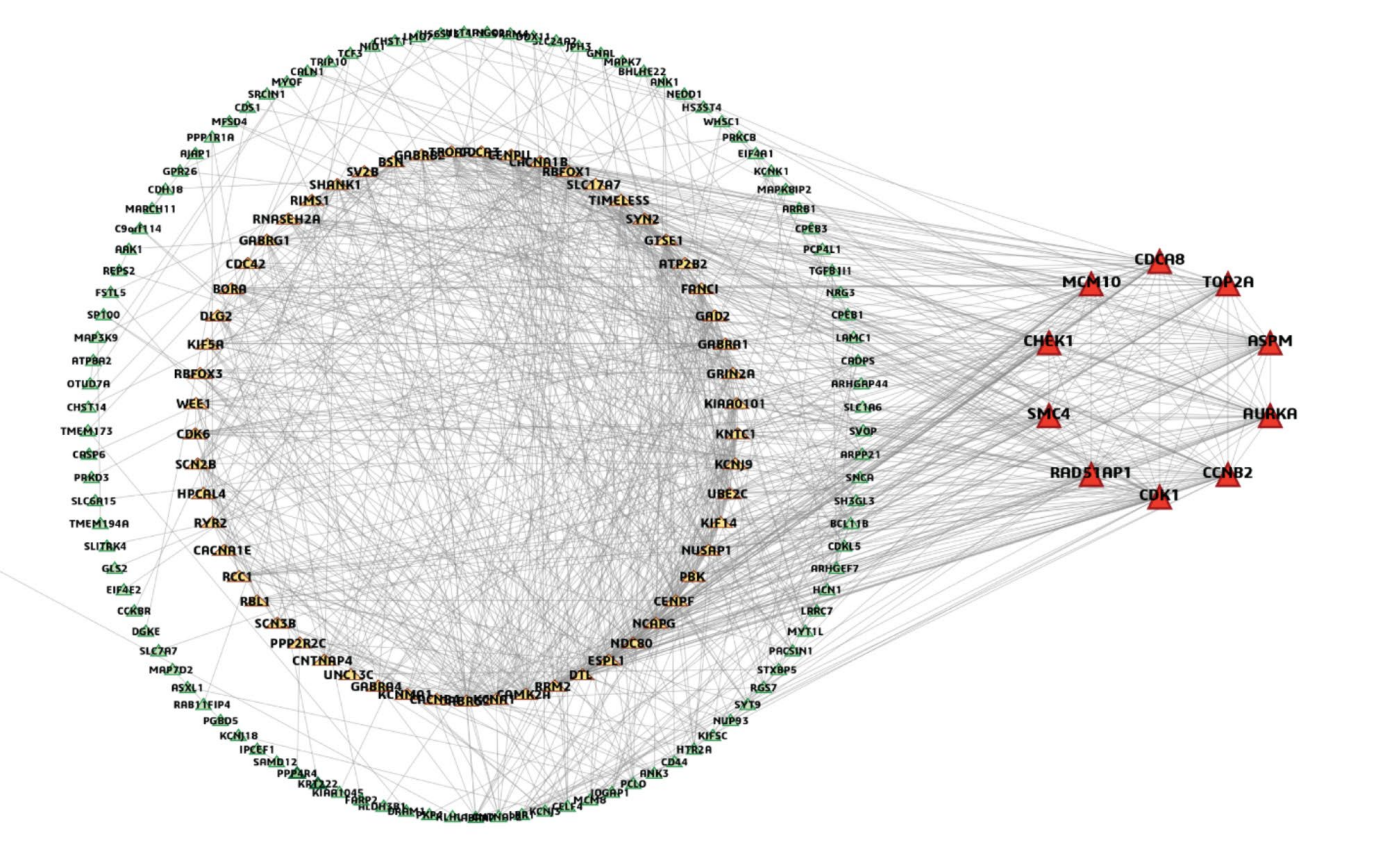
PPI network study of cDEGs. Green nodes indicate lower interactions, yellow nodes indicate medium interactions and red nodes indicate higher interactions (KGs).

The PPI network was built using cDEGs, resulting in a network composed of 172 nodes and 977 edges. Top rank 10 kg (ASPM, CCNB2, CDK1, AURKA, TOP2A, CHEK1, CDCA8, SMC4, MCM10, and RAD51AP1) were selected from the PPI network by applying six topological measures (Fig. 5 and Table S6). Further we conducted module analysis with the cDEGs to locate key genes (KGs) in the clusters. Two modules were detected. Notably, all the KGs, detected by six topological measures were found in ‘module 1’ (Figure S5).

### Verification of differential expression patterns of KGs using independent datasets

At first, we verified the differential expression patterns of KGs in two independent databases (GTEx and TCGA) that combinedly contains 207 normal and 163 GBM samples through the box plot analysis (Figure S6.). We found that all KGs are upregulated that support our findings. To assess the prediction performance of KGs, we developed a Random Forest (RF) based prediction model using 60% samples as train data. The rest 40% data was used as test data. We also considered another independent test dataset from NCBI database with accession ID GSE50161. For both the test datasets, we constructed the ROC curves (Figure S7) and calculated some performance scores (AUC, TPR, TNR, and Accuracy) (Table S7). The performance of KGs in both prediction models was found to be strong with an AUC > 0.989 and ACC > 0.92.

### Functional enrichment analysis of KGs with the GO-terms and KEGG pathways

For 10 kg, we carried out GO and KEGG pathway analysis. Here we took into account the most important GO terms from each cellular component (CC), biological process (BP), molecular function (MF), and KEGG pathways with P-value < 0.05 (Table S8).

### KGs regulatory network analysis

The TFs and miRNAs networks were used to examine the regulators of KGs. We chose the top two TFs (GATA2, FOXC1) according to two topological measures, betweenness and degree with cutoff of 177 and 7 respectively as they play most prominent role in transcriptional level of the KGs (Figure S8-A). By employing the exact topological measures method, we chose the top five miRNAs (hsa-mir-16-5p, hsa-mir-34a-5p, hsa-mir-205-5p, hsa-mir-124-3p, and hsa-mir-147a) with betweenness and degree cutoff of 1225 and 10 respectively (Figure S8-B).

### DNA methylation analysis of KGs in GBM

DNA methylation is an epigenetic process that controls the expression of genes^82^. DNA methylation allows researchers to uncover biomarkers for early detection, disease prognosis, and potential therapeutic targets. DNA methylation of essential genes helps researchers understand the regulatory mechanisms behind critical cellular processes and their disruption in disease, making it a vital aspect of genomic research^83^. Therefore, we examined DNA methylation status of KGs in GBM by MethSurv. We observed that except SMC4, the other nine KGs had significant CpG sites (p-value of ≤ 0.05) (Table S9). Additionally, ULCAN was also utilized to visualize the methylation status of the KGs in GBM. From Box whisker plot (Figure S9) it was found that almost all the KGs are hypomethylated in both GBM and Normal samples according to β-values ranging from 0 (completely unmethylated) to 1 (highly methylated). Some of KGs (TOP2A, CCNB2, CDK1, and, MCM10) showed almost no significant methylation differences between GBM and normal samples (β-values almost same). Rest of the KGs showed significant methylation differences between GBM and normal samples (lower β-values in GBM compared to normal samples).

### Immune infiltration level analysis of KGs

The tumor microenvironment (TME) is a complex environment composed of different stromal components including immune cell along with the tumor cells^84^. To predict the infiltration of immune cells in GBM by the TIMER algorithm, we assessed the correlations between the expression levels of the KGs and the levels of infiltration of six immune cells (CD8 + T cell, B cell, CD4 + T cell, dendritic cell, neutrophil, and macrophage) (Figure S10). The findings indicate that the expression of KGs has a strong and positive relationship with the infiltration level of CD8 + T cell (0.12 ≤ Rho ≤ 0.35) and B cell (0.10 ≤ Rho ≤ 0.275) and weak and negative relationship with the infiltration level of CD4 + T cell (-0.15 ≥ Rho≥-0.39), Neutrophil (-0.19 ≥ Rho≥-0.298), Macrophage (0.11 ≥ Rho≥-0.016) and Dendritic cell (0.15 ≥ Rho≥-0.16). This result could help to discover potential immunotherapy for GBM.

### KGs-guided drug repurposing by molecular docking

**Fig. 6:**
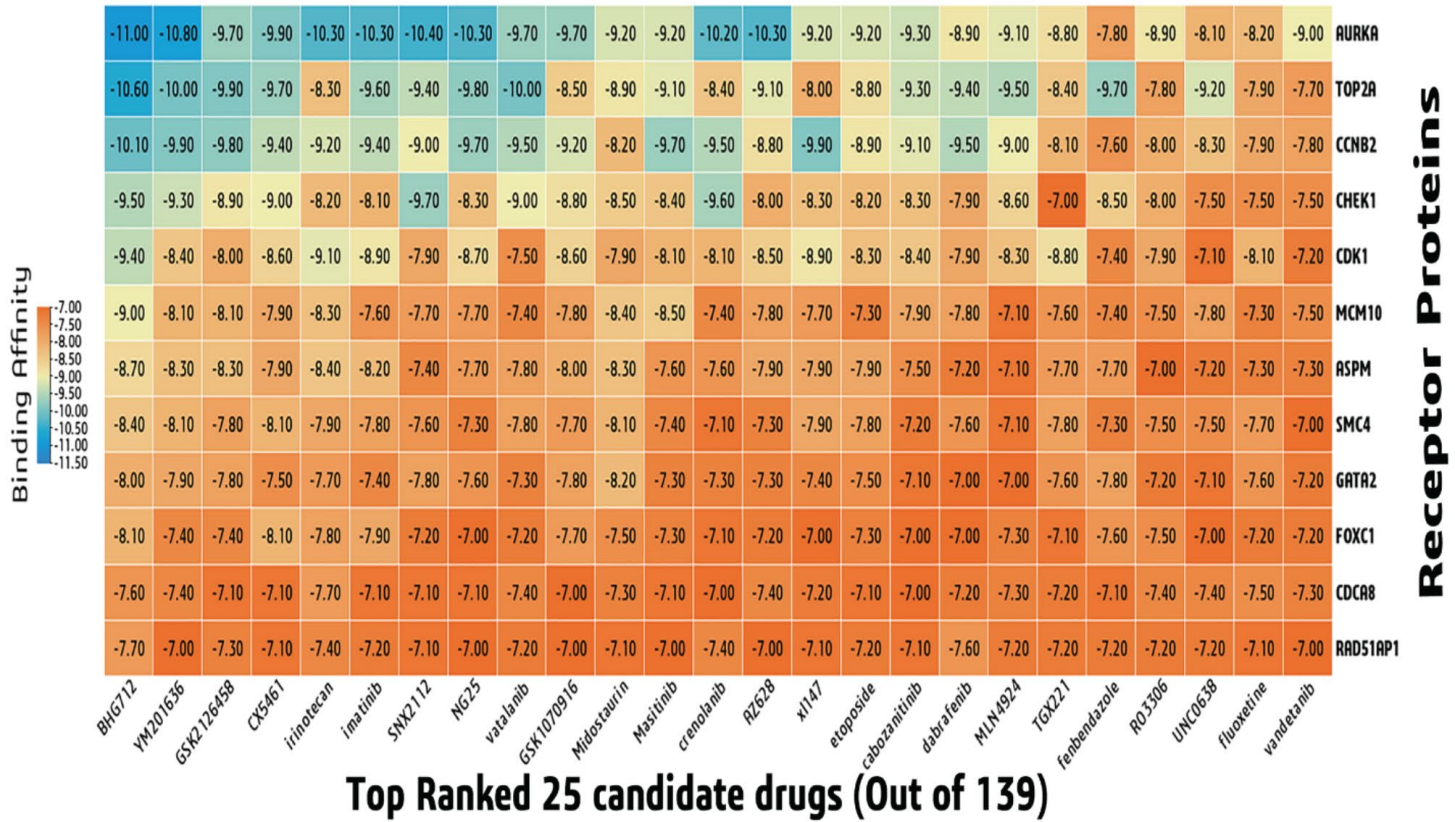
Binding affinities between receptor proteins and drug agents.

To explore KGs-guided repurposable drug molecules, we performed molecular docking between KGs mediated receptors and candidate drug molecules. The 3D structures of seven receptors (CDK1, AURKA, TOP2A, CHEK1, CDCA8, SMC4, and GATA2) were taken from the Protein Data Bank (PDB) using the following PDB codes: 6GU6, 2J4Z, 1ZXM, 1ZLT, 2KDD, 4U4P, 6ZFV. Four targets (CCNB2, MCM10, RAD51AP1, FOXC1) were obtained from the “AlphaFold Protein Structure Database” (AF-O95067-F1, AF-Q7L590-F1, AF-Q96B01-F1, AF-Q12948-F1) using their corresponding UniProt IDs, O95067, Q7L590, Q96B01, Q12948, respectively. The remaining one receptor (ASPM, uniport id Q8IZT6) was obtained from swiss model after homology modeling using template (P62295.1.A). Out of 139 drugs, the top-ranked 25 potential drugs were considered as potential drugs because all of them exhibited significant binding affinity (BA) < -7.0 (kcal/mol) after docking (Fig. 6). In terms of potential treatments for GBM, these 25 lead compounds appear promising.

### Evaluation of drug-likeness and ADMET properties

Based on the Lipinski rule of five (ROF) we found that, out of top-ranked 25 drugs, 9 drugs (SNX2112, vatalanib, crenolanib, MLN4924, TGX221, fenbendazole, RO3306, fluoxetine and, vandetanib) violates no ROF (Table S10). Then, the ADME/T Properties of the 9 drugs were examined through various parameter. The water solubility (ESOL) score of all the 9 drugs were computed based on the LogP value. We found that the logP value off all the drugs were in range of -5.9 to -3.4 (poorly soluble < − 6 < moderately < − 4 < soluble) which indicates that they are water soluble^68^ (Table 2). A compound’s blood-brain barrier (BBB) permeability index indicates how likely it is to pass the BBB (a physiological barrier between the blood and the central nervous system). Compounds having a logBB > 0.3 can penetrate the BBB and logBB < -1 are poorly distributed to the brain. Though our study was based on GBM (one of the malignant brain cancers), it is very important that the drug molecules should cross BBB to exhibit its function inside the brain^85,86^. We found that 5 drugs (Fluoxetine, Vatalanib, TGX221, Fenbendazole and, RO3306) are more likely and 2 drugs (Crenolanib and Vandetanib) are less likely to possess the capability to penetrate the BBB. According to the Drug-Likeness and ADMET analysis of 25 drug molecules we concluded that, four compounds (Fluoxetine, Vatalanib, TGX221 and RO3306) could be the potential drugs for GBM. Table S11 displayed the interactions profile of the four drugs with the top ranked potential receptor AURKA.

**Table 2:** ADMET profile of top-ranked nine drugs.

Drug compounds (LogP)	Absorption		Desorption		Metabolism	Excretion	Toxicity	
	Caco2 Permeability	HIA (%)	BBB (Permeability)	CNS (Permeability)	CYP3A4	TC	LC_50_	LD_50_ (mole/kg)
**Fluoxetine (-4.36)**	1.764	91.371	0.501	-1.32	Yes	0.694	1.25	2.87
**Vatalanib (-5.20)**	1.431	95.00	0.313	-1.78	Yes	-0.086	-0.45	3.19
**TGX221 (-3.46)**	1.194	98.08	0.471	-2.02	Yes	0.547	-0.56	2.88
Fenbendazole (-4.08)	0.867	88.17	0.176	-2.12	No	0.734	-0.13	2.46
**RO3306(-4.77)**	1.40	89.1	0.399	-1.80	Yes	-0.137	0.47	2.50
Vandetanib (-5.89)	1.40	90.74	-0.037	-2.20	Yes	0.548	0.44	2.89
SNX2112(-5.06)	0.69	89.84	-1.29	-2.45	Yes	-0.029	1.35	2.38
Crenolanib (-5.01)	1.36	87.98	-0.26	-2.35	Yes	0.987	-1.79	2.26
MLN4924(-3.62)	0.524	84.75	-1.21	-3.26	Yes	0.560	2.51	2.38

### Molecular dynamic (MD) simulations with the top-ranked drug-target complexes

**Fig. 7:**
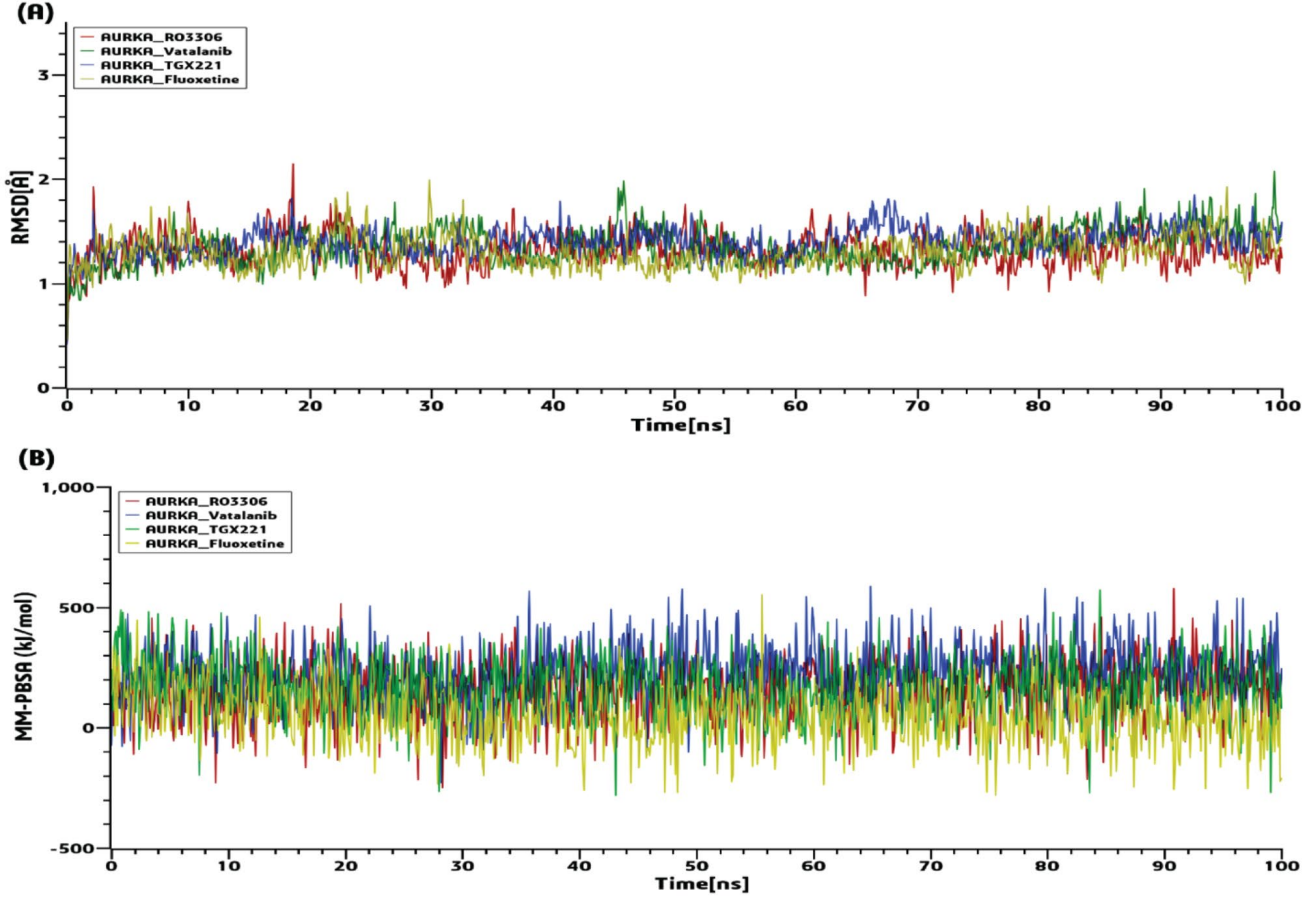
100ns MD simulation results for the top four complexes. **(A)** The RMSDs’ periodic progression. **(B)** MM-PBSA binding free energy (kJ/mol).

After docking and ADME/T analysis, we selected four drugs - Fluoxetine, Vatalanib, TGX221 and RO3306 as four candidate drug molecules. Therefore, 100 ns MD-based MM-PBSA simulations were run on the top-ranked receptor (AURKA) and the four drug complexes (AURKA-RO3306, AURKA-Vatalanib, AURKA-TGX221, and AURKA-Fluoxetine) to evaluate their stability. All complexes showed a minor fluctuation in Cα backbone but remained stable rest of the simulation. The RMSD (root mean square deviation) related to the proposed receptor (AURKA) was displayed in Fig. 7(A). The estimated RMSDs ranged from 0.41 to 2.15. The AURKA complexes’ average RMSDs were 1.32, 1.35, 1.40, and 1.30, respectively. The RMSD of all the complexes raised slightly between 0 and 10 ns and remained stable till 100 ns. From the graph it can be clearly interpreted that all the complexes were structurally stable. The four complexes’ binding energies were calculated for the MM-PBSA shown in Fig. 7(B).

## Discussion

Due to the heterogeneity of GBM, a high mortality and fatality rate still persists. So, it is essential to identify GBM causing key molecular signatures for diagnosis, prognosis and therapies. In this research, we considered nine microarray gene-expression datasets to discover GBM causing key genes (KGs). At first, we detected 220 overlapping DEGs between GBM and control samples by using machine learning (ML) and WGCNA approaches. Then the top-ranked 10 DEGs (ASPM, CCNB2, CDK1, AURKA, TOP2A, CHEK1, CDCA8, SMC4, MCM10, and RAD51AP1) were identified as the KGs via PPI network and module analysis. (Fig. 5, Table S6, Figure S5). Previous individual studies also reported some of these KGs as the GBM causing KGs. Among them, the key gene ‘Cyclin-dependent kinase 1 (CDK1)’ is a part of a group of cell cycle-regulating kinases. Its primary role involves overseeing the transition of the BP-term ‘G2/M phase’ (Table S7) of the cell cycle, and facilitating the initiation of mitosis through its interaction with cyclin B^87^. The G2/M phase has a potential role in the growth of GBM tumors^88,89^ Also, the inhibition of CDK1 through knockdown experiments resulted in a noteworthy reduction in the proliferation of GBM cells, specifically in U-87MG and U-251MG cell lines indicating that CDK1 is essential for the proliferation of GBM cells^90^. Aurora kinase A (AURKA) gene is a member of the MF-term “serine/threonine kinase”, and its activation plays a crucial role in governing cell division by controlling the process of mitosis^91^. It plays an important role in the development and spread of solid tumors, including glioblastomas^92^. A serine/threonine-specific protein kinase called checkpoint kinase 1 (CHEK1), also known as CHK1, controls the cell cycle checkpoint response and the DNA damage response^93^. It was also enriched in different KEGG pathways including cell cycle. Numerous CHK1 inhibitors were shown to interact with numerous MEK1/2 inhibitors to eradicate a variety of primary human glioblastoma isolates^94^. Moreover, other KGs (e.g., ASPM, CDCA8, MCM10 etc.) play an important role in developing GBM and associated with different biological process, molecular function and pathways ^12,95–97^. The expression analysis from the independent NCBI, TCGA and GTEx databases confirmed the differential expression patterns of KGs (Figure S6, S7). Some TFs and miRNAs were detected as the key transcriptional and post-transcriptional regulators of KGs by the gene regulatory network analysis (Figures S8) which might play a crucial role in the development of GBM. The DNA methylation study indicated that all the KGs (except SMC4) had CpG sites (Table S9) which might play an important role in GBM development.

DNA methylation analysis showed that most of the KGs are hypomethylated for which they become more active as oncogene^98^. From several studies, we found that these oncogenes are associated with the development of different cancers including GBM^91,99–104^. Tumor immunotherapy has emerged as a new area of study for tumors in recent years. In order to better understand the tumor microenvironment, more research has been concentrated on the immune cells’ penetration into tumor tissues^105^. We examined the association between the expressions of KGs and immune infiltrating cell types (CD8 + T and CD4 + T cell, B cell, neutrophil, dendritic cell and macrophage) in GBM and found their significant association in GBM progression and development (Figure S10). It has been found that CD8 + T cell infiltration positively correlates with the survival rate of patients with GBM^106^ and B cells were discovered to infiltrate in GBM^107^. However, it was found that almost all the KGs were poorly correlated with macrophage infiltration. It could indicate that higher gene expression is associated with lower immune cell infiltration, suggesting that these genes may contribute to immune evasion or suppression^108,109^. Some of these genes were also found to be weakly correlated with macrophage infiltration in previous studies^12,110,111^. Additionally, study showed that GBM located in the temporal lobe exhibited the highest levels of macrophage infiltration, while those in the frontal lobes had significantly lower levels of macrophage infiltration^112^.

We investigated potent drugs for the treatments against GBM and found four drugs (Fluoxetine, Vatalanib, TGX221 and RO3306) displayed favorable profiles. Among the identified candidate drugs, Fluoxetine has FDA approval for the therapy of major depressive disorder, as indicated by their Drug Bank (DB) database (DB accession number DB00472). It is one of the most prescribed selective serotonin reuptake inhibitor (SSRI), which increase the intracellular [Ca2+], thereby triggering apoptosis in gliomas^113^. Currently, research is being done on vatalanib to treat oral angiogenesis (accession numbers DB04879). The metastasis of GBM U87 cells after receiving CPI444 and vatalanib via a nanocarrier (GO-PEG) was significantly reduced^114^. It was discovered that TGX-221 prevented glioblastoma cells from migrating and invading, which allowed it to prevent cell growth and trigger apoptosis^115^. At dosages that inhibit CDK1, it was discovered that RO-3306 had no standalone cytotoxic impact but sensitized a number of GBM cells to Temozolomide (TMZ)^87^. Finally, the stability of the top-docked complexes (AURKA-RO3306, AURKA-Vatalanib, AURKA-TGX221 and AURKA-Fluoxetine) was assessed through molecular dynamics (MD)-based MM-PBSA simulation. The results indicated that these complexes exhibited consistent and stable behavior. Among the proposed drugs, TGX-222 and RO-3306 have not been approved yet and require further evaluation through wet-lab based experiments before clinical trial for the treatment of GBM.

## Conclusion

This study identified GBM-causing 10 key genes (KGs) from nine transcriptomics datasets by using both supervised and unsupervised learning approaches. The association of KGs with GBM was also confirmed by some independent datasets/databases. The KGs-set enrichment analysis with GO-terms and KEGG pathways revealed some crucial biological process (DNA replication, G2/M transition of mitotic cell cycle), molecular functions (protein serine/threonine kinase activity, single-stranded DNA binding) and pathways (p53 signaling pathway, Cell cycle) associated with GBM. The KGs regulatory network analysis revealed two TFs (FOXC1 and GATA2) and five miRNAs (hsa-mir-16-5p, hsa-mir-34a-5p, hsa-mir-205-5p, hsa-mir-124-3p, hsa-mir-147a) as the transcriptional and post-transcriptional regulators. DNA methylation studies also showed that most of the KGs are hypomethylated which indicates their oncogenic activities. The infiltration level analysis of KGs revealed that, KGs are significantly associate with different tumor infiltrates immune cells such as, CD8 T cell, CD4 T cell, B cell, neutrophil, macrophage and dendritic cell (DC) of GBM. Four top-ranked potential drugs (Fluoxetine, Vatalanib, TGX221 and RO3306) were identified by molecular docking, drug-likeness and ADMET analysis. Therefore, the output of this study may play a vital role for diagnosis and therapies of GBM.

## Electronic supplementary material

Below is the link to the electronic supplementary material.

Supplementary Material 1

## Data Availability

The datasets analyzed in this study are freely available at the following links https://www.ncbi.nlm.nih.gov/geo/query/acc.cgi?acc=GSE9171, https://www.ncbi.nlm.nih.gov/geo/query/acc.cgi?acc=GSE15824, https://www.ncbi.nlm.nih.gov/geo/query/acc.cgi?acc=GSE19728, https://www.ncbi.nlm.nih.gov/geo/query/acc.cgi?acc=GSE32374, https://www.ncbi.nlm.nih.gov/geo/query/acc.cgi?acc=GSE35493, https://www.ncbi.nlm.nih.gov/geo/query/acc.cgi?acc=GSE43289, https://www.ncbi.nlm.nih.gov/geo/query/acc.cgi?acc=GSE68848, https://www.ncbi.nlm.nih.gov/geo/query/acc.cgi?acc=GSE86574, https://www.ncbi.nlm.nih.gov/geo/query/acc.cgi?acc=GSE104291, https://www.ncbi.nlm.nih.gov/geo/query/acc.cgi?acc=GSE50161.
