# Supplementary material for: Discovery of key molecular signatures for diagnosis and therapies of glioblastoma by combining supervised and unsupervised learning approaches
Source: Sci Rep. 2024 Nov 11;14:27545. doi: 10.1038/s41598-024-79391-2 (PMC11554889; doi:10.1038/s41598-024-79391-2)
Supplement: Supplementary file 1 — Supplementary Material 1 [file 41598_2024_79391_MOESM1_ESM.docx]

**Supplementary File**

**Discovery of key molecular signatures for diagnosis and therapies of glioblastoma by combining Supervised and Unsupervised Learning Approaches**

Arnob Sarker^1^, Md. Abdul Aziz^1^, Md. Bayazid Hossen^2^, Md. Manir Hossain Mollah^3^, Al-Amin ^4^ and Md. Nurul Haque Mollah^2^*

^1^Department of Biochemistry and Molecular Biology, University of Rajshahi, Rajshahi 6205, Bangladesh ^2^Bioinformatics Lab (Dry), Department of Statistics, University of Rajshahi, Rajshahi 6205, Bangladesh ^3^Department of Physical Sciences, Independent University, Bangladesh (IUB), Dhaka, Bangladesh ^4^Department of Zoology, University of Rajshahi, Rajshahi 6205, Bangladesh

***Corresponding Author:** E-mail: Md. Nurul Haque Mollah ([mollah.stat.bio@ru.ac.bd](mailto:mollah.stat.bio@ru.ac.bd))

| **Supporting Items** | **Pages** |
| --- | --- |
| Section S1: | **4** |
| **Supplementary Figures** |  |
| **Figure S1.** Network topology analysis for soft-thresholding powers selection. The left panel depicts the Scale-free fit index for different powers (β). The right panel depicts the Mean connectivity analysis for various soft-thresholding powers (β). The power when the correlation is required to reach 0.8 is used as the β value. In case of (A) GSE68848 it was 11, (B) GSE86574 it was 18 and (C) GSE104291 it was 15. | 5 |
| **Figure S2.** Clustering of module eigengene for merging close modules. Cut height of module eigengene was set to 0.1 for (A) GSE68848, 0.15 for (B) GSE86574 and, 0.30 for (C) GSE104291. | 6 |
| **Figure S3.** The correlation values and associated P-values (in parenthesis) were used to indicate the module-trait relations, and a wide range of colors were used to represent them. Module Eigengenes (MEs) are displayed in the rows, and the column indicates trait (GBM). Blue, yellow, green, magenta, pink, brown modules from (A) GSE68848; turquoise, light-yellow, brown, green, pink, royal-blue from (B) GSE86574; and saddle-brown, red, pale-turquoise, brown modules from (C) GSE104291; had significant correlation with trait. | 7 |
| **Figure S4.** Scatter plots to visualize the correlation between gene significance (GS) and module membership (MM) of the significant modules. Each dot represents a gene. Higher correlation indicates that these modules are suitable for identifying the DEGs associated with GBM. DEGs were identified from the modules (**(A)** GSE68848 – (1-6); **(B)** GSE86574 – (1-6); and **(C)** GSE104291 – (1-4)) by setting the criteria GS ≥ 0.7 and MM ≥ 0.8. Higher GS and MM indicate highly interconnected genes in the module. | 8-9 |
| **Figure S5.** Module analysis of cDEGs indicating that all the KGs (sky-blue colored octagonal nodes) belong to the “module 1”. | 10 |
| **Figure S6.** Boxplots show the difference of KGs-expressions between GBM and control groups. Red and black indicates GBM and control groups, respectively. | 11 |
| **Figure S7**. ROC curves based on the RF-based prediction models with KGs. | 12 |
| **Figure S8.** Regulatory network of the KGs. **(A)** Network of TFs-KGs interaction. KGs are marked as sky-blue colored hexagonal shape. Top ranked two TFs are marked as red colored octagonal shaped. Rest of the TFs are marked as orange color with circular shape. **(B)** Network of TFs-miRNAs interaction. KGs are represented as pink colored octagonal shaped. The highest ranked five miRNAs are indicated as larger and rest of the miRNAs are shown as relatively smaller sky-blue colored circular shape. | 13 |
| **Figure S9.** Promoter methylation status of KGs with Box whisker plot. Red and blue boxplots indicating the methylation expression patterns of the KGs in GBM and normal samples respectively | 14 |
| **Figure S10.** The scatter plot showed the relationship between the expression of KGs and immune infiltrating levels of CD8+ T, CD4+ T cell, B cell, neutrophil, dendritic cell and macrophage in GBM. | 15-16 |
| **Supplementary Tables** |  |
| **Table S1.** Collection of brain cancer related drug molecules from different sources. | 17-18 |
| **Table S2.** Features of the selected modules | 19 |
| **Table S3**. List of upregulated and downregulated DEGs by Random Forest (RF) and Support Vector Machine (SVM) approach**es.** | 20-21 |
| **Table S4.** List of DEGs by WGCNA. | 22-23 |
| **Table S5.** List of common DEGs (cDEGs) between two approaches (ML and WGCNA). | 23-24 |
| **Table S6**. List of key genes (KGs) from PPI network based on different topological measures | 24 |
| **Table S7.** Test performance scores of the RF-based prediction model with the cutoff at FPR < 10% | 24 |
| **Table S8.** The top significantly (p-value<0.05) enriched GO functionals and KEGG pathways by KGs. | 25-27 |
| **Table S9.** Methylation Status of the KGs in GBM by MethSurv. | 27 |
| **Table S10**. Lipinski rule of 5. The drugs highlighted in bold font satisfied all the five rules. | 28 |
| **Table S11**. The top ranked receptor protein (AURKA) and top 4 lead compounds (Fluoxetine, Vatalanib, TGX221 and RO3306) based on ADMET and docking results. The third column displays the 3D structure of receptor protein AURKA along with potential therapeutics. The neighboring residues (within 4 Å of the drug) are displayed in the fourth column by the 2D Schematic representation of the receptor-drug interactions. The final column displayed the interaction types and amino acids of the receptor proteins which took part in those interactions. | 29 |
| **References** | 30 |

**Supplementary Section S1.**

**Lemma:** Variance of differentially expression (DE) pattern is greater than variance of equally expression (EE) pattern for *i*th gene, that is $s_{i, DE}^{2}>s_{i, EE}^{2}.$

**Proof:** Let $g_{ij}$ represents the expression of *i*th gene with *j*th sample (*i*=1, 2, ..., *n; j=*1, 2,..., *m*), then $s_{i}^{2}$ can be wrirren as $s_{i}^{2}=\frac{1}{m}\sum_{j=1}^{m} \left( g_{ij}-\bar{g}_{i} \right)^{2},$ where $\bar{g}_{i}=\frac{1}{m}\sum_{j=1}^{m} g_{ij}$. If *i*th gene is differentially expressed (DE) between case and control groups, that is $\bar{g}_{i, 1}=\frac{1}{m_{1}}\sum_{j=1}^{m_{1}} g_{ij}\neq\frac{1}{m_{2}}\sum_{j=m_{1}+1}^{m_{2}} g_{ij}=\bar{g}_{i,2},$ (*m*=$m_{1}+m_{2}),$then $s_{i}^{2}$ can be expressed as

$$s_{i, DE}^{2}=\frac{1}{m}\sum_{j=1}^{m} \left( g_{ij}-\bar{g}_{i} \right)^{2}=\frac{1}{m}\left[ m_{1}\left( s_{i, 1}^{2}+d_{i, 1}^{2} \right)+m_{2}\left( s_{i, 2}^{2}+d_{i, 2}^{2} \right) \right]\ldots\ldots\ldots\ldots\ldots\ldots\ldots\ldots(2)$$

where

$s_{i,k}^{2}=\frac{1}{m_{k}}\sum_{j=1}^{m_{k}} \left( g_{ij}-\bar{g}_{i,k} \right)^{2}, \bar{g}_{i,k}= \frac{1}{m_{k}}\sum_{k=j}^{2} g_{ij}, \bar{g}_{i}=\frac{1}{m}\sum_{k=1}^{2} m_{k}\bar{g}_{i,k}, \mathrm{and} d_{i, k}=\bar{g}_{i,k}-\bar{g}_{i}\neq0,$since $\bar{g}_{i, 1}\neq\bar{g}_{i,2},$

If *i*th gene is equally expressed (EE) between case and control groups, then $\bar{g}_{i,1}=\bar{g}_{i,2}$, which implies $\bar{g}_{i}=\bar{g}_{i,1}=\bar{g}_{i,2}$, then $d_{i, k}=0$ (*i*=1, 2, ..., *n; k*=1, 2). Then equation (2) becomes,

$$s_{i, EE}^{2}=\frac{1}{m}\sum_{j=1}^{m} \left( g_{ij}-\bar{g}_{i} \right)^{2}=\frac{1}{m}\left( m_{1}s_{i, 1}^{2}+m_{2}s_{i, 2}^{2} \right)\ldots\ldots\ldots\ldots\ldots\ldots\ldots\ldots(3)$$

Thus, from equations 2 and 3, it is seen that $s_{i, DE}^{2}> s_{i, EE}^{2}.$ ]

**Supplementary Figures.**


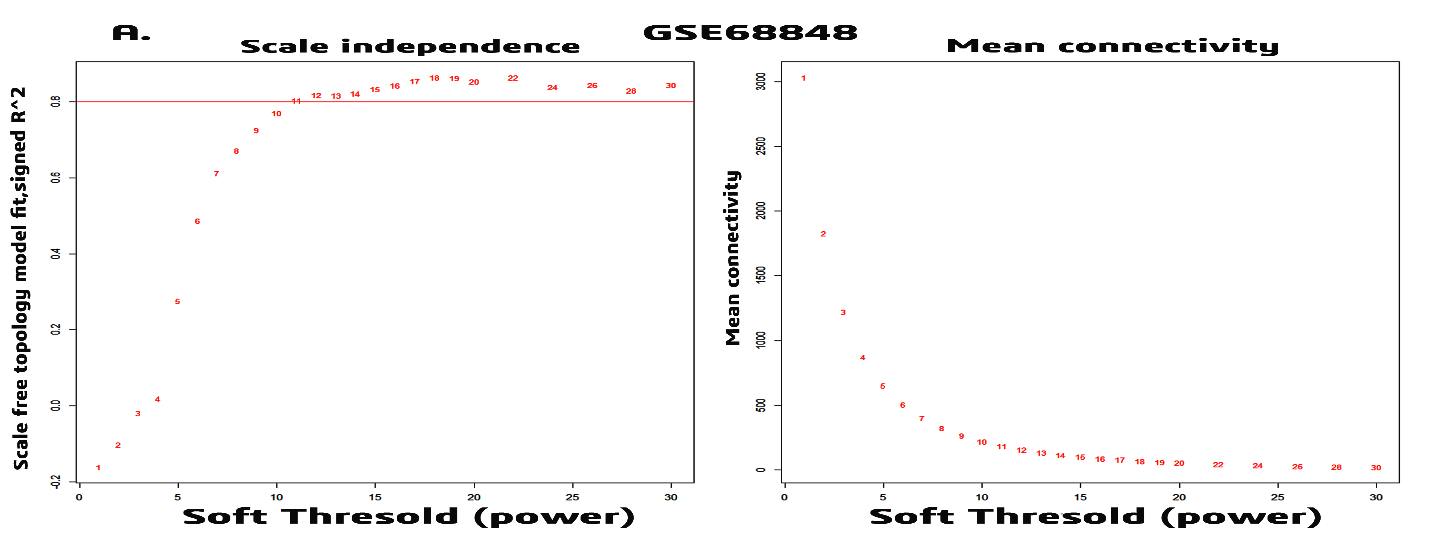

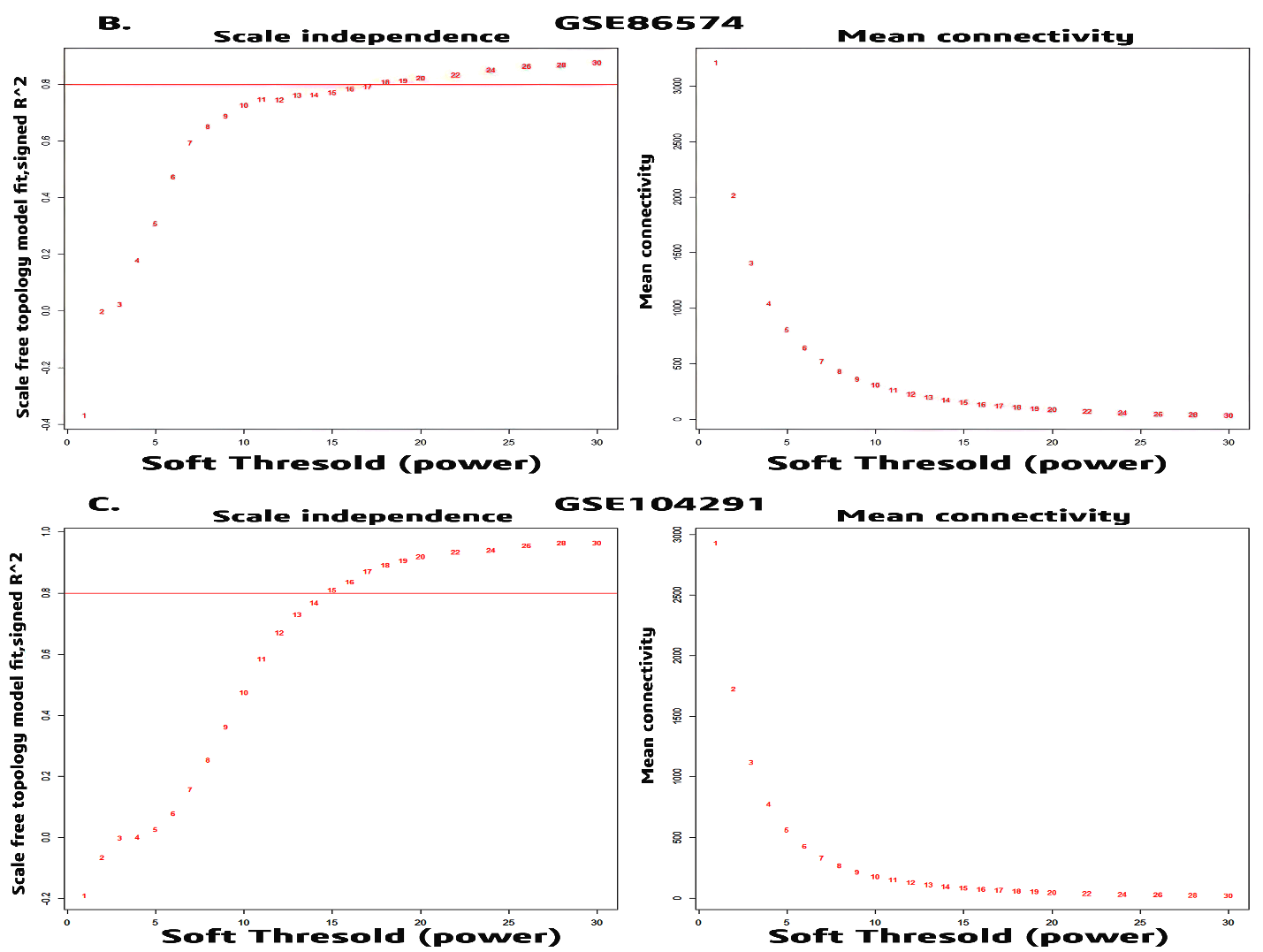


**Figure S1.** Network topology analysis for soft-thresholding powers selection. The left panel depicts the Scale-free fit index for different powers (β). The right panel depicts the Mean connectivity analysis for various soft-thresholding powers (β). The power when the correlation is required to reach 0.8 is used as the β value. In case of (A) GSE68848 it was 11, (B) GSE86574 it was 18 and (C) GSE104291 it was 15.


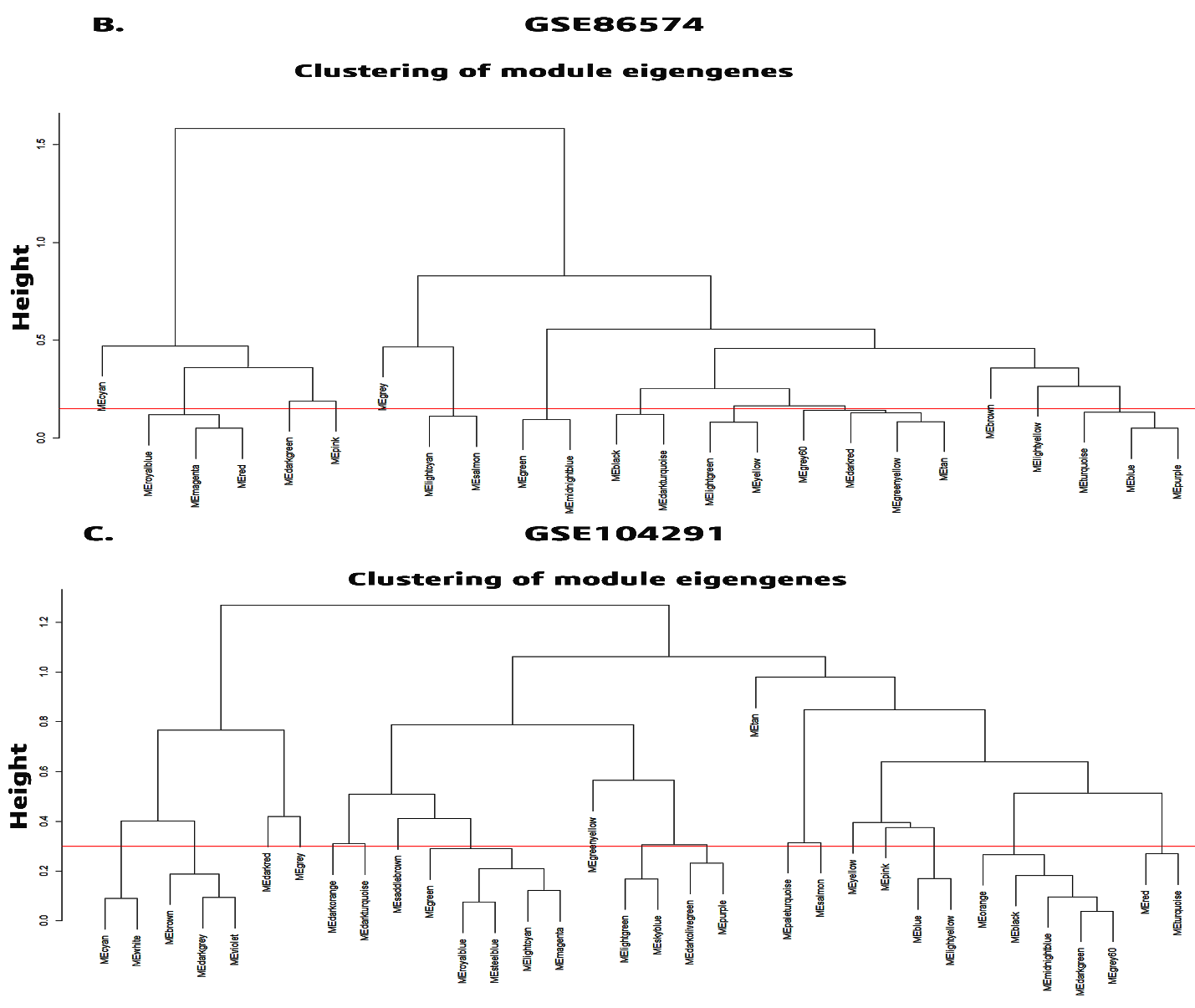

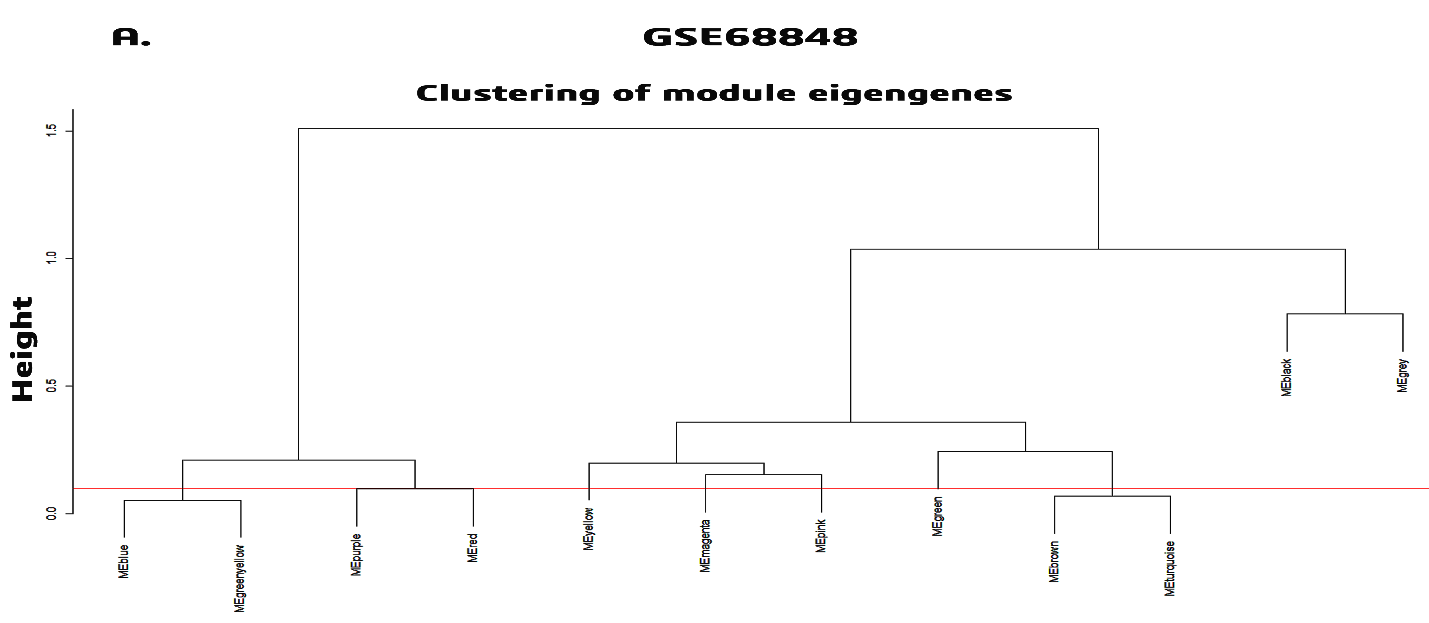


**Figure S2.** Clustering of module eigengene for merging close modules. Cut height of module eigengene was set to 0.1 for (A) GSE68848, 0.15 for (B) GSE86574 and, 0.30 for (C) GSE104291.


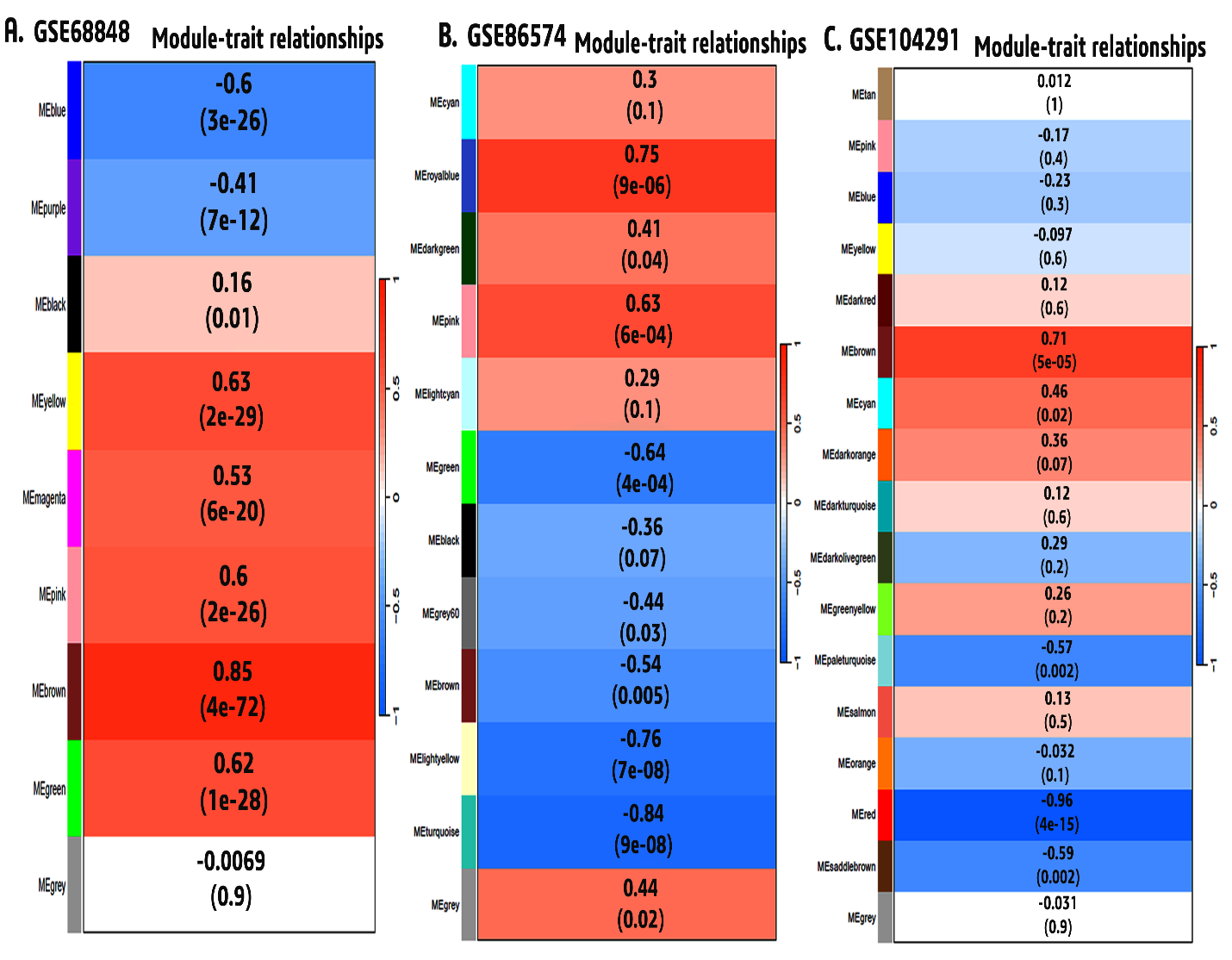


**Figure S3.** The correlation values and associated P-values (in parenthesis) were used to indicate the module-trait relations, and a wide range of colors were used to represent them. Module Eigengenes (MEs) are displayed in the rows, and the column indicates trait (GBM). Blue, yellow, green, magenta, pink, brown modules from (A) GSE68848; turquoise, light-yellow, brown, green, pink, royal-blue from (B) GSE86574; and saddle-brown, red, pale-turquoise, brown modules from (C) GSE104291; had significant correlation with trait.


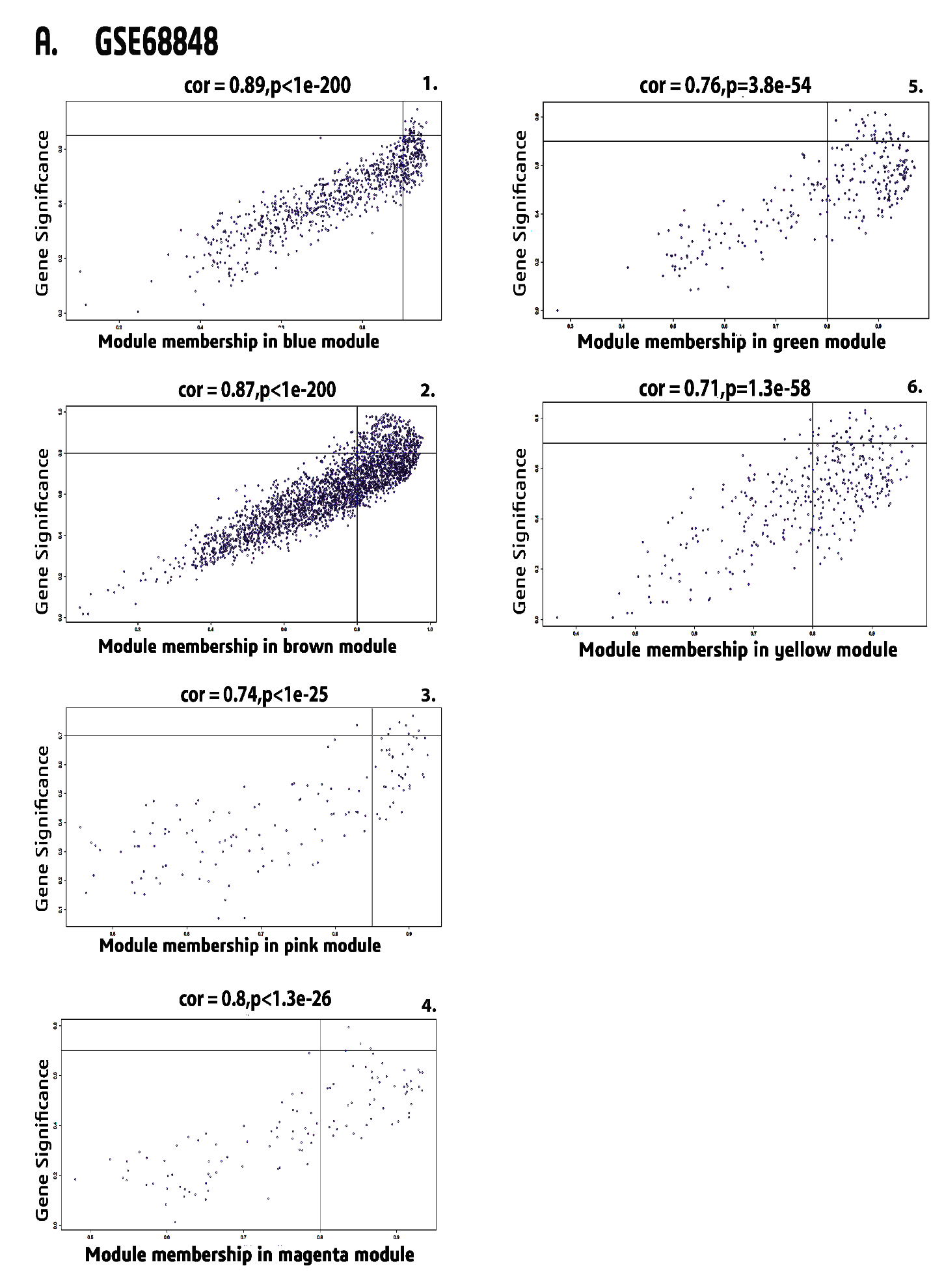


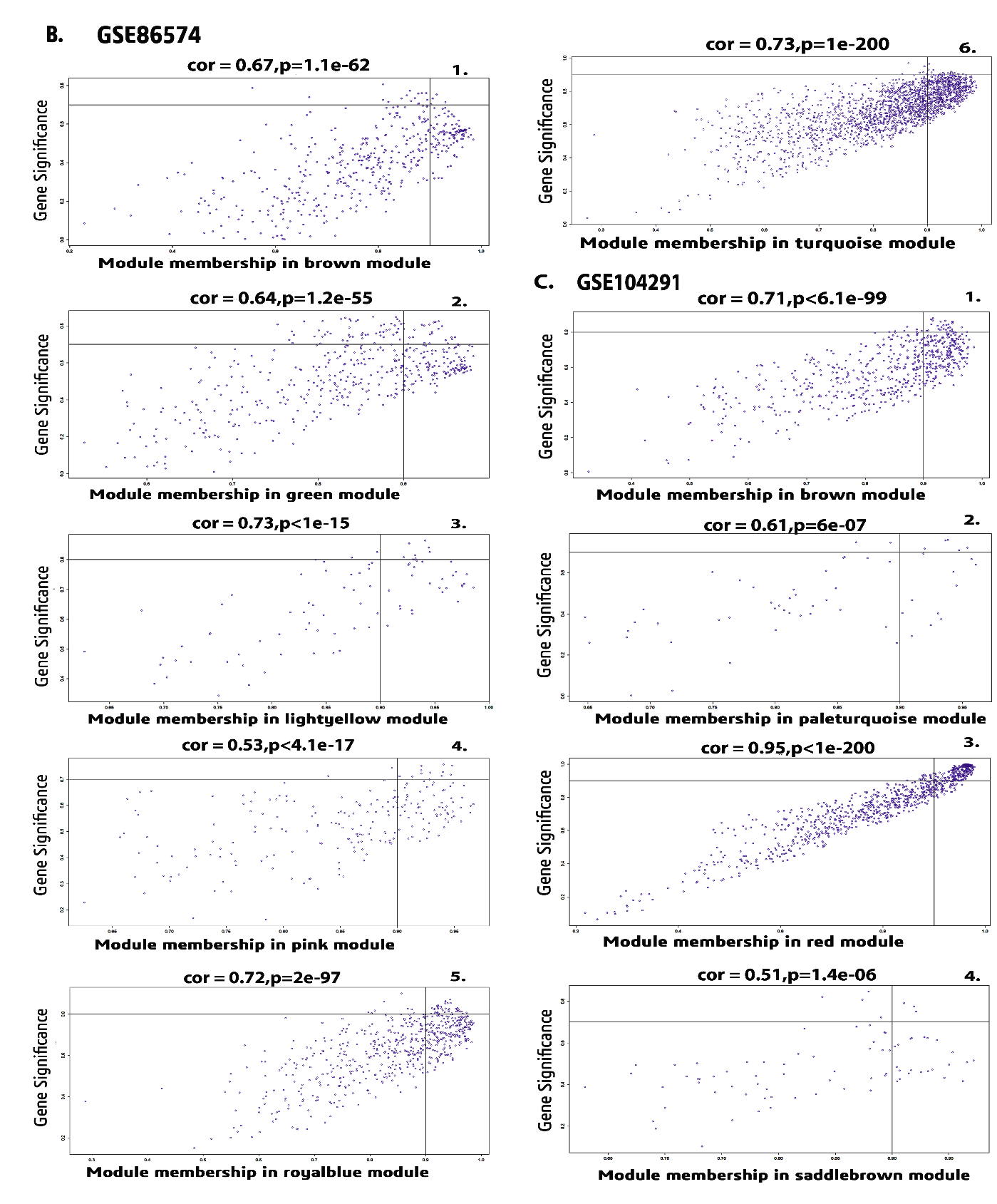


**Figure S4.** Scatter plots to visualize the correlation between gene significance (GS) and module membership (MM) of the significant modules. Each dot represents a gene. Higher correlation indicates that these modules are suitable for identifying the DEGs associated with GBM. DEGs were identified from the modules (**(A)** GSE68848 – (1-6); **(B)** GSE86574 – (1-6); and **(C)** GSE104291 – (1-4)) by setting the criteria GS ≥ 0.7 and MM ≥ 0.8. Higher GS and MM indicate highly interconnected genes in the module.


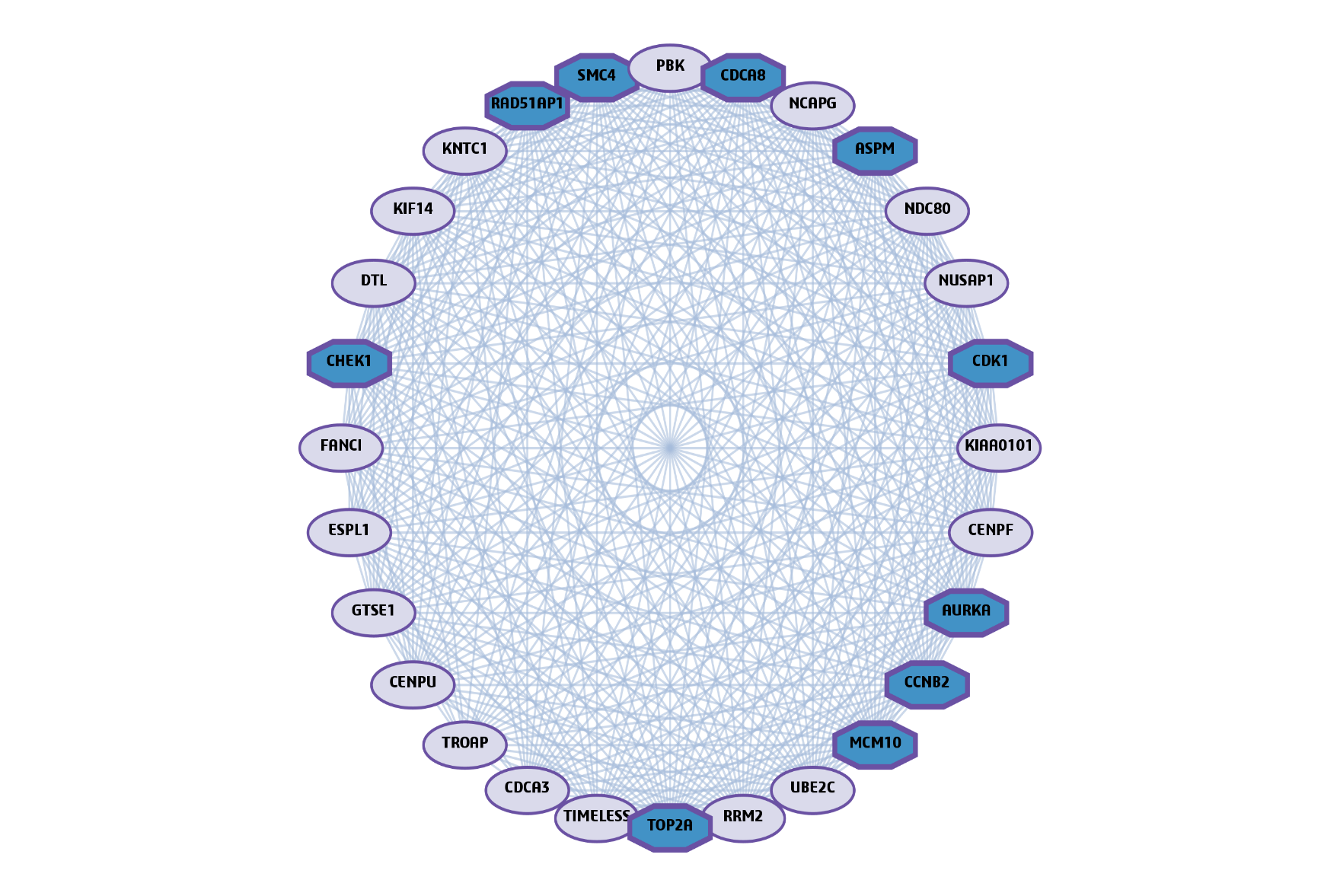


**Figure S5.** Module analysis of cDEGs indicating that all the KGs (sky-blue colored octagonal nodes) belong to the “module 1”.


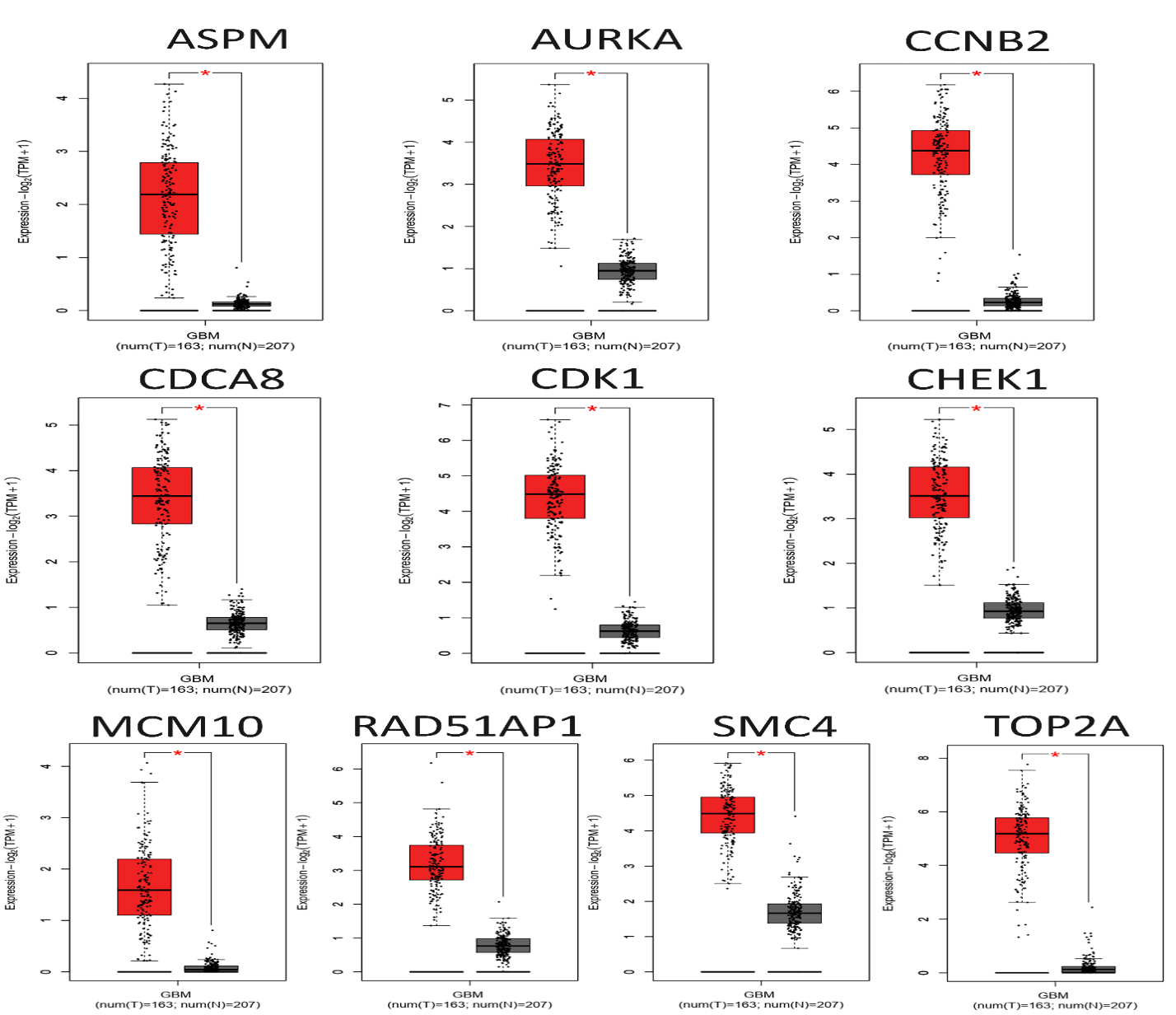


**Figure S6.** Boxplots show the difference of KGs-expressions between GBM and control groups. Red and black indicates GBM and control groups, respectively.


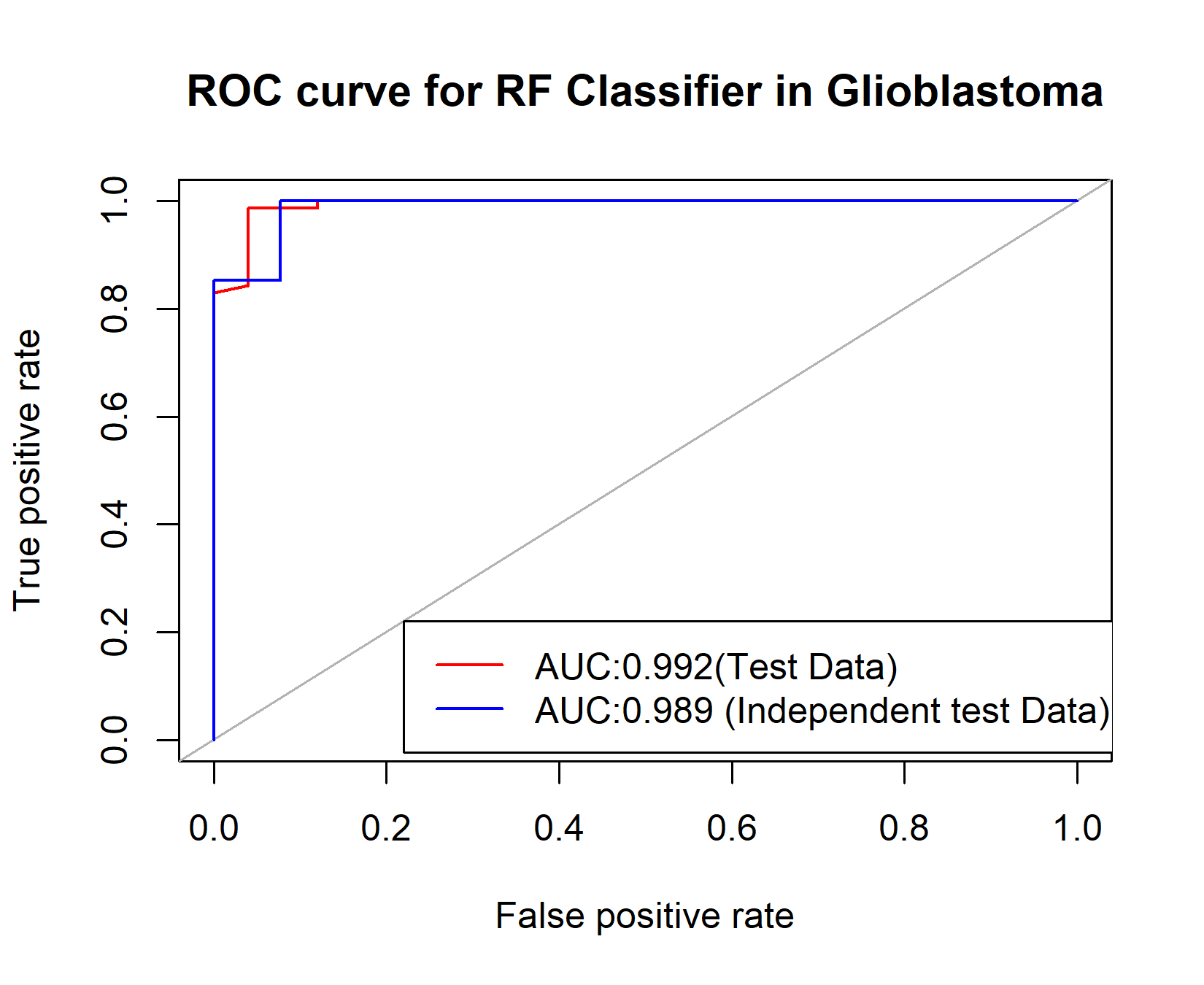


**Figure S7**. ROC curves based on the RF-based prediction models with KGs.


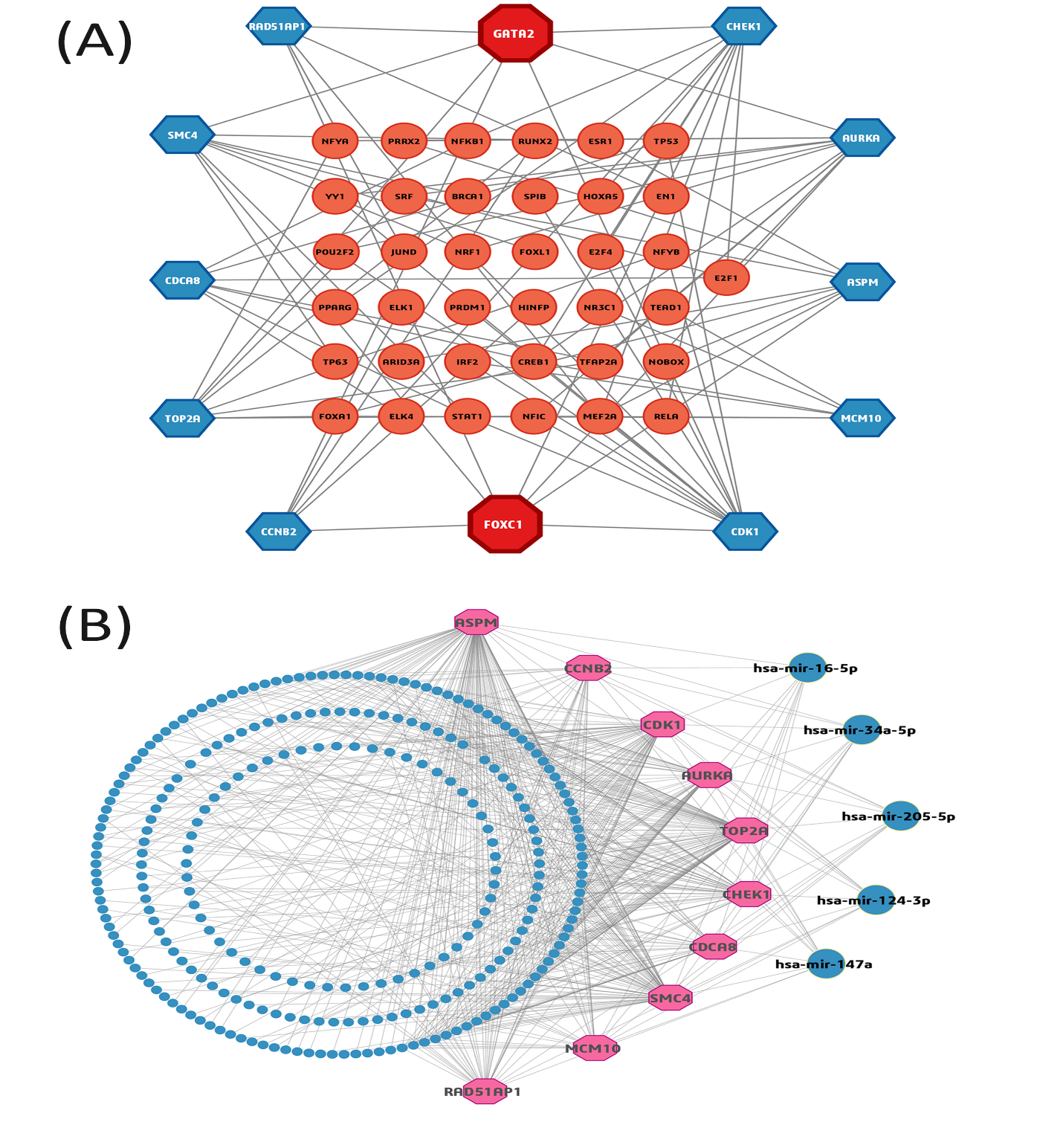


**Figure S8.** Regulatory network of the KGs. **(A)** Network of TFs-KGs interaction. KGs are marked as sky-blue colored hexagonal shape. Top ranked two TFs are marked as red colored octagonal shaped. Rest of the TFs are marked as orange color with circular shape. **(B)** Network of TFs-miRNAs interaction. KGs are represented as pink colored octagonal shaped. The highest ranked five miRNAs are indicated as larger and rest of the miRNAs are shown as relatively smaller sky-blue colored circular shape.


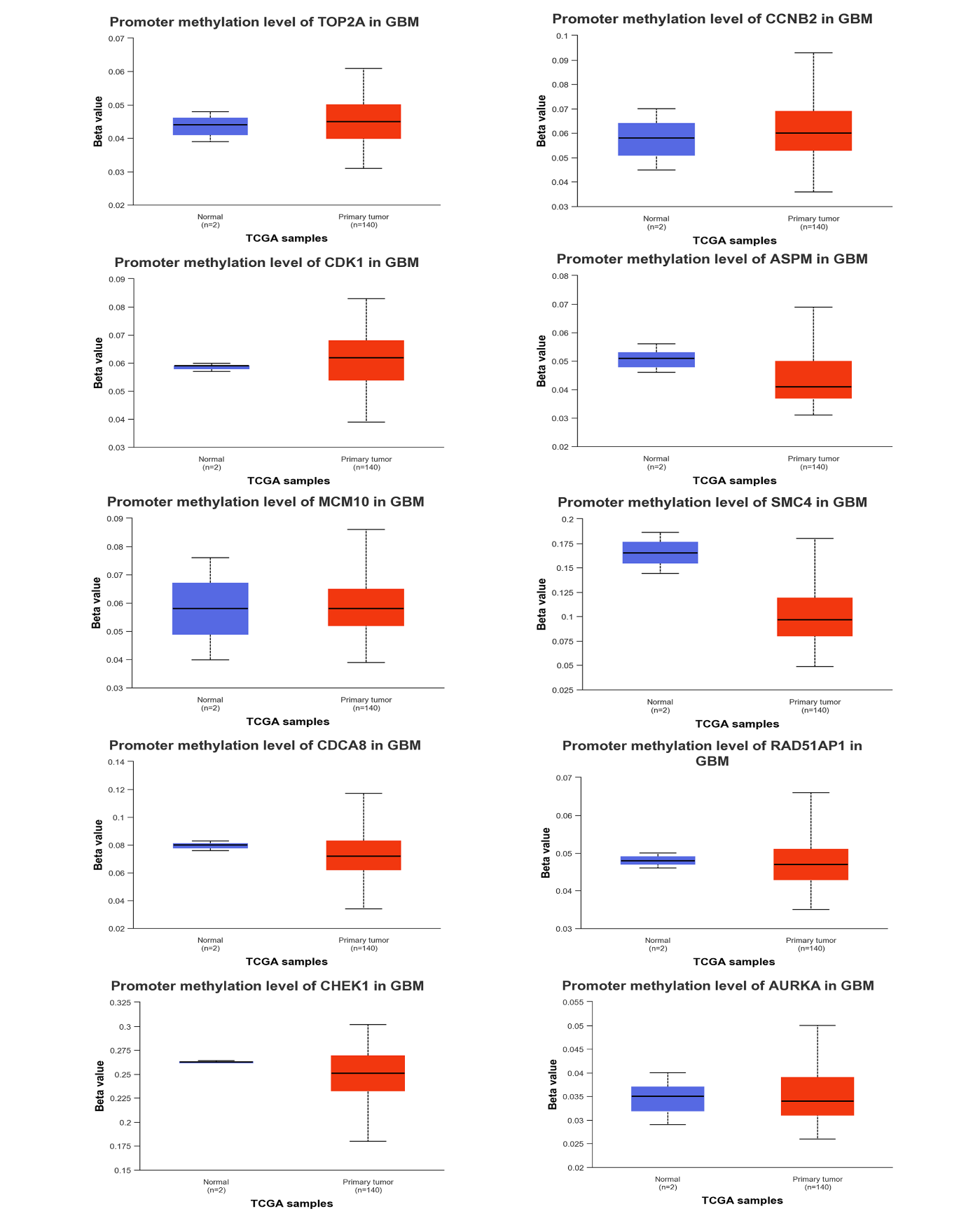


**Figure S9.** Promoter methylation status of KGs with Box whisker plot. Red and blue boxplots indicating the methylation expression patterns of the KGs in GBM and normal samples respectively


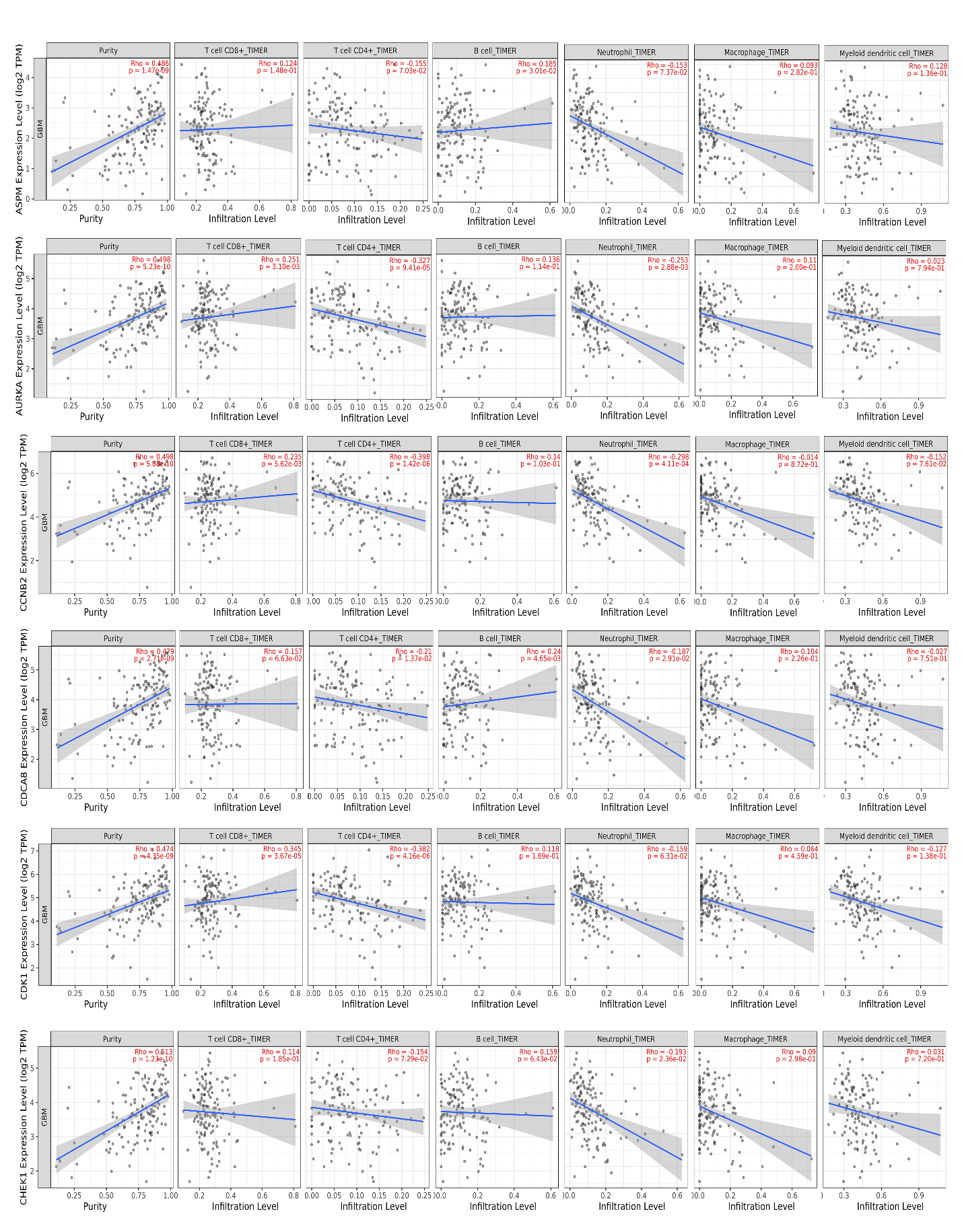


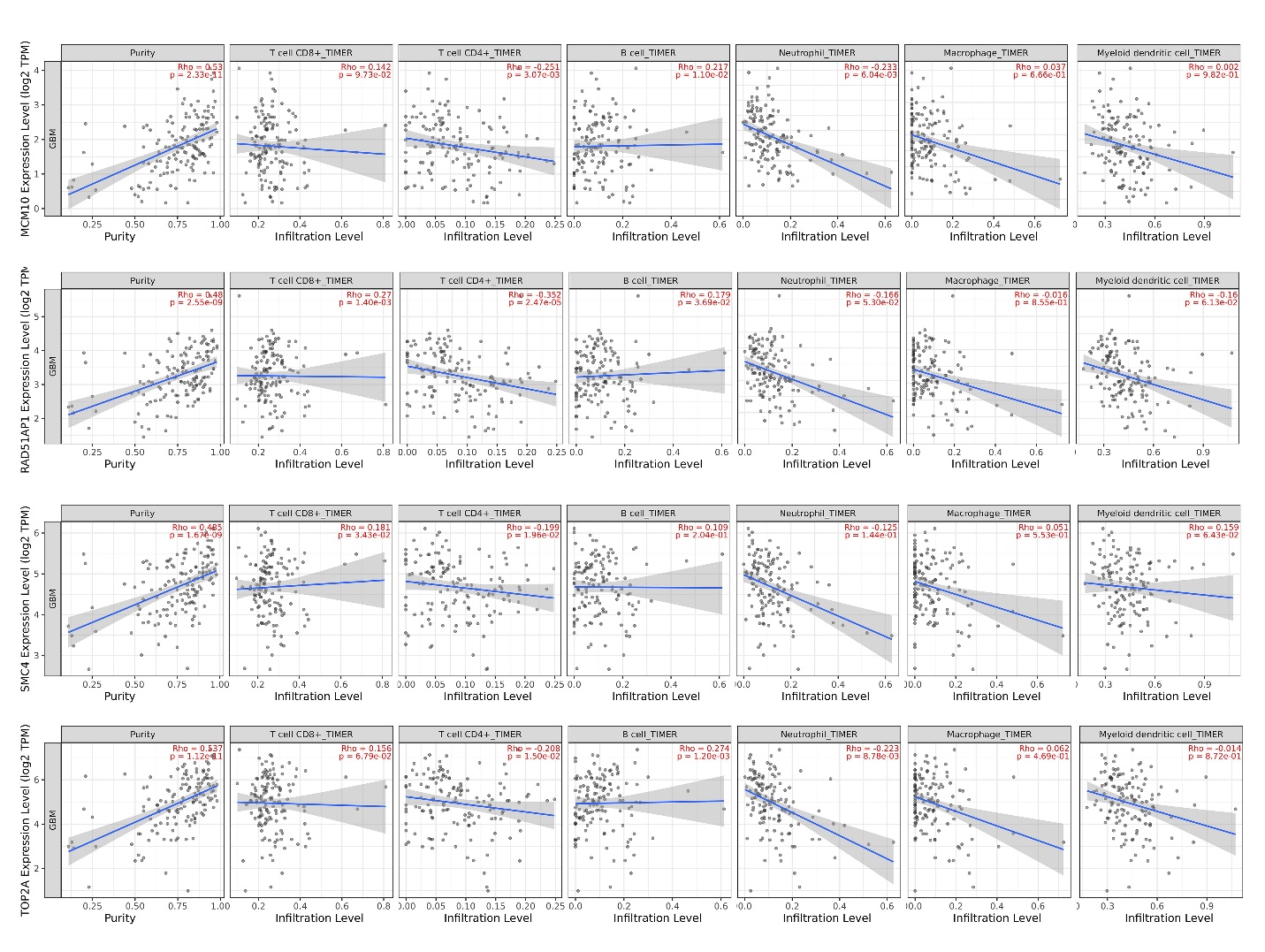


**Figure S10.** The scatter plot showed the relationship between the expression of KGs and immune infiltrating levels of CD8+ T, CD4+ T cell, B cell, neutrophil, dendritic cell and macrophage in GBM.

**Supplementary Tables.**

**Table S1.** Collection of brain cancer related drug molecules from different sources.

| Reference | Drugs |
| --- | --- |
| (Staedtke et al., 2016) ^1^ | Procarbazine, CCNU/Lomustine, Vincristine, temozolomide, bevacizumab , erlotinib, gefitinib, lapatinib, cetuximab, pazopanib, Dacomitinib, Afatinib, cetuximab, panitumumab, nimotuzumab, AMG 595, Sym004, ABT-414, imatinib mesylate, sunitinib, sorafenib, pazopanib, dasatinib, crenolanib, cabozanitinib, rilotumumab, onartuzumab, cediranib, pazopanib, vandetanib, vatalanib, everolimus, sapanisertib, buparlisib, Oncothyreon, XL147, Vemurafenib, dabrafenib, trametinib, selumetinib, decitabine, 5-azacytidine, Dichloroacetate, Gabapentin, vorinostat, valproic acid, panobinostat, Rindopepimut, HSPPC-96, ICT-107, nivolumab, pidilizumab, pembrolizumab, ipilimumab, pidilizumab, |
| (Juillerat-Jeanneret, 2008) ^2^ | Doxorubicin, Paclitaxel, Cisplatin, Iirinotecan, Methotrexate, Temozolomide |
| (Zhuo et al., 2019) ^3^ | valproic acid (VPA), fluoxetine (FLX), escitalopram oxalate, saha (ARP),  Aripiprazole, Clozapine, Fluphenazine, Olanzapine, Quetiapine, Thioridazine |
| (Kim et al., 2011) ^4^ | suberoylanilide hydroxamic acid (SAHA), erlotinib |
| (Wainwright et al., 2012) ^5^ | TP-38, Bevacizumab, CRM-107, Everolimus, Imatinib, Sirolimus, Resveratrol, Temozolomide, Temsirolimus, IL-13 toxin, IL-4 PE |
| (Sontheimer & Bridges, 2012) ^6^ | Sulfasalazine |
| (Newton, 2000) ^7^ | Temozolomide, marimastat, batimastat, AG3340, BAY 12-9566, Irinotecan, angiostatin, endostatin, suramin, thalidomide, TNP-470, marimistat, SU-6668, retinoids, paclitaxel, BCNU |
| (Laquintana et al., 2009) ^8^ | Carmustine, lomustine, nimustine, anthracyclines, platinum(II)complexes, paclitaxel, etoposide, irinotecan, topotecan, methothrexate, procarbazine, lomustine, vincristine, Temozolomide, Gefitinib, Lapitinib, Erlotinib, Vatalanib, Imatinib, Temsirolimus, Bortezomid, Bevacizumab |
| (Benjamin, 2019) ^9^ | rolipram, apremilast, folumilast |
| (Fung et al., 2019) ^10^ | Bevacizumab, bkm120, erlotinib, pembrolizumab, trametinib, selumetinib, buparlisib, dactolisib, everolimus |
| (Bahmad et al., 2021) ^11^ | valproic acid, Mebendazole, Metformin, diltiazem, verapamil, SMV, pioglitazone, metformin, phenformin, Atorvastatin, LVS, tideglusib, kenpaullone, ibuprofen, diclofenac, Chlorpromazine, Thioridazine, Triflupherazine, Fluoxetine, imipramine, amitriptyline, fluvoxamine, fenbendazole, MBZ |
| DSigDB (Yoo et al., 2015) ^12^ | trifluridine, troglitazone, methotrexate, Fulvestrant, ciclopirox, deferoxamine, monobenzone, LUCANTHONE, genistein, resveratrol, curcumin, thalidomide, benzo[a]pyrene, etoposide, nocodazole, 5-azacytidine, CUPRIC, 5-Fluorouracil, doxorubicin, Zoledronic, vinblastine, calcitriol, paclitaxel, SB, rapamycin, PD, quercetin, estradiol, HMN-176, testosterone |
| GSCALite (C. J. Liu et al., 2018) ^13^ | CP466722, OSI−027, Navitoclax, TAK−715, Bleomycin, 5−Fluorouracil, Methotrexate, WH−4−023, Dasatinib, GSK269962A, Y−39983, Docetaxel, (5Z)−7−Oxozeaenol, Bortezomib, MG−132, MLN4924, XMD8−85, KIN001−135, CX−5461, QL−XII−47, BIX02189, BMS345541, JW−7−24−1, KIN001−236, KIN001−260, NG−25, SNX−2112, TL−1−85, XMD13−2, YM201636, 17−AAG, QL−X−138, TPCA−1, I−BET−762, Crizotinib, TAE684, Midostaurin, Pazopanib, Masitinib, BHG712, QL−XI−92, TG101348, XMD14−99, WZ3105, UNC0638, VX−680, Genentech, Ispinesib_Mesylate, GSK1070916, NPK76−II−72−1, KIN001−270, RO−3306, THZ−2−49, THZ−2−102−1, AT−7519, PHA−793887, CAL−101, BX−912, GSK2126458, KIN001−244, ZSTK474, GSK690693, KIN001−102, PI−103, PIK−93, TGX221, AZ628, CI−1040, SB590885, Dabrafenib, TL−2−105, PD−0325901, RDEA119, selumetinib, Trametinib, Belinostat, CAY10603, AR−42, CUDC−101, Tubastatin, Vorinostat, Afatinib, Cetuximab, Erlotinib, Gefitinib, Lapatinib, SB, CHIR−99021, FK866, Phenformin |

| **Table S2.** Features of the selected modules | | | | | | |
| --- | --- | --- | --- | --- | --- | --- |
| Dataset | Modules | Correlations | p-value | GS | MM | Filtered genes |
| GSE104291 | Brown | 0.71 | 5e-05 | 0.8 | 0.9 | 30 |
|  | Pale turquoise | -0.60 | 0.002 | 0.8 | 0.9 | 5 |
|  | Red | -0.96 | 4e-15 | 0.9 | 0.9 | 254 |
|  | Saddle brown | -0.61 | 0.002 | 0.7 | 0.9 | 3 |
| GSE68848 | Brown | 0.85 | 4e-72 | 0.8 | 0.8 | 191 |
|  | Pink | 0.60 | 2e-26 | 0.7 | 0.85 | 7 |
|  | Magenta | 0.61 | 6e-20 | 0.7 | 0.8 | 3 |
|  | Green | 0.62 | 1e-26 | 0.7 | 0.8 | 32 |
|  | Yellow | 0.63 | 2e-29 | 0.7 | 0.8 | 37 |
|  | Blue | 0.60 | 3e-26 | 0.7 | 0.9 | 28 |
| GSE86574 | Royal blue | 0.75 | 9e-06 | 0.8 | 0.9 | 35 |
|  | Pink | 0.63 | 6e-04 | 0.7 | 0.9 | 11 |
|  | Green | -0.64 | 4e-04 | 0.7 | 0.9 | 24 |
|  | Brown | -0.60 | 0.005 | 0.7 | 0.9 | 4 |
|  | Light yellow | -0.76 | 7e-06 | 0.8 | 0.9 | 9 |
|  | Turquoise | -0.84 | 9e-08 | 0.9 | 0.9 | 22 |

**Table S3**. List of upregulated and downregulated DEGs by Random Forest (RF) and Support Vector Machine (SVM) approach**es.**

| Upregulated DEGs |
| --- |
| DTL, GNB4, ZFHX3, PLEKHG2, AGAP2-AS1, ADAM12, BRCA1, GFPT1, CENPF, ANKFN1, ABCC3, BMP1, CENPA, ASPM, CDCA2, CDK1, PARPBP, MKI67, ERI1, C15orf39, NRP1, IQGAP1, SLC29A1, CPXM1, TBL1X, KIF23, PABPC1L, GABPA, SDC1, ANXA2P1, LPP, LEFTY2, RDH10, HOXD10, TRMT5, TMEM256-PLSCR3, SINHCAF, FPR3, ITGA2, TBX2, OSR2, ASXL1, SMC4, ITGB8, TFAP2A, RNASEH2A, WDR53, MCM8, SERPINH1, EMILIN2, TTC38, TCF3, ADGRE2, LINC02381, FSTL1, IBSP, ATL3, LTBP1, PCLAF, HOXC6, GPX7, CKLF, ALDH3B1, BNC2, NCAPG, TNFAIP8, CMTM3, ADAMTS6, SYTL4, ARHGAP18, HMMR, NAGA, DDX11, CCNB2, FAM114A1, P3H2, RAB42, UTP15, ANTXR1, CDK6, GM2A, STEAP3, NFE2L3, IGFBP5, PTGFRN, TP53, NID1, CSPG4, GPX8, ATP6V0A2, MPZL1, ANGPTL2, PAFAH2, ZNF367, IPO4, TRIM21, PLVAP, IL17RC, IFT56, GLB1L, NRP2, H4C12, SP100, KDELR3, FABP7, EIF4A1, POFUT1, FBLIM1, CAPS, RIPK1, UGGT1, FIRRM, DNAI4, SYDE1, ELF4, THOC2, ARSJ, RRM2, PGAP1, TP53I3, SERPINA5, CCDC80, MCM10, NDC80, MUC1, CHD9, SYTL3, PRDM5, GTSE1, POLE2, CHEK1, BCL3, COMMD4, WEE1, CHST14, CA3, LRIG3, RCN3, SRPX2, FARP2, NLRC5, SGSH, TRPM8, PRKD3, TBX15, RHOC, BUB1, THADA, SERTAD3, CNPY4, UNC93B1, MMP19, SHOX2, HELLS, IKBIP, LAMC1, ASF1B, EIF4E2, AFG2A, NIBAN1, KIAA0040, ELMOD2, CENPI, SPATA6, TEAD4, NEMP1, PI3, SP140L, BATF2, TOP2A, PLEKHA4, ZGRF1, HELZ2, PMM2, CYP3A5, SNX20, BORA, CDCA7, STING1, TGFB1I1, CD44, AURKB, CENPU, LRR1, TRIM5, TRIOBP, DRAM1, DOT1L, ANG, IL13RA1, SLC35F2, VIM, NOD1, CD276, PBK, ATAT1, TNFRSF10B, HAS2, FOXD1, NEDD1, CDCA8, STIL, MSR1, ARSD, MKS1, RAD51, COL5A1, MBOAT1, RBBP8, ADAL, NUF2, QSER1, EMP1, ZNF606, MAP3K7CL, PSMB8, LRRC69, EIF4EBP1, AURKA, PCGF1, DHODH, CDC20, EEF1AKMT3, ITGB3, MSH5-SAPCD1, ITPRIP, PLEK2, HOXB3, ANTXR2, SLC7A7, PARP12, HOXA3, PLA2G2A, GEN1, DNAH9, TFDP1, CDCA3, CAVIN1, TCF19, NUSAP1, MIR4435-2HG, PSMC3IP, GPR107, MTFR2, IQGAP2, FLJ32255, SLAMF8, RRBP1, RNF213, TTK, DENND2D, CD163, FBXO4, MAP3K20, RAD51AP1, ITGA4, PLK4, ACTMAP, MICALL1, POLQ, ZIK1, TOR1AIP2, NFIA-AS2, CARD8, KIAA1549, PIMREG, CDT1, KIF14, LOX, PALLD, MIR3945HG, CEP89, SLC30A6, KIFC1, IGKV1D-13, CKAP2L, CEP55, PRKAA1, FOLR1, SCAMP2, TMEM45A, SERINC2, E2F7, ESPL1, WWTR1, THBS3, PLAGL2, NSD2, TBXA2R, ARHGEF40, SCARF2, MYOF, NAT1, SPOUT1, TRIP10, BCL2L12, CSTA, ZNF217, PTTG3P, CLEC5A, DEPDC1, MTHFD2L, ZCCHC10, TRAF4, TRPS1, PMP22, DLGAP5, MAPK7, ALKBH8, ISG20, E2F8, MR1, EPHB2, UBE2C, TNIP2, DHRSX, MARVELD3, HAS3, MCM5, CDC25A, E2F3, ZNF234, COL22A1, TRABD, POLR1E, RECQL, TIMELESS, C21orf62, AJUBA, MCM7, SPC25, TMEM71, PABIR2, MTMR11, FAM83D, H2AX, OTP, KIF15, CRNDE, CHST11, OLFML2A, WDR5B, HOXB7, SPAG4, CDIN1, FANCI, EXO1, ANXA2P2, STC2, NDUFA10, TROAP, OAS1, COL6A1, WDFY2, ZNF625-ZNF20, KLRK1-AS1, DDR2, L3HYPDH, LAMP1, ATAD3A, HOTAIRM1, KNTC1, KIF18B, RBL1, FNDC3B, HJURP, STAMBP, GLI2, RCC1, FXYD5, POLR1A, BRCA2, CTPS2, TFPI, BARD1, FAM111A, CASP6, CDC45, SOX6, PARP9, S100A10 |
| Downregulated DEGs |
| KCNH3, MFSD4A, GLS, SULT4A1, CACNA1E, GPR26, NETO1, ZDHHC18, MAPRE2, ITPR1, KCNJ3, LY86-AS1, ANK3, EPS15, RAB18, TMEM130, PRSS3, UNC13C, BDNF, KCNN1, GABRA1, DUSP8, CNTFR-AS1, TBR1, AJAP1, TBC1D30, RFPL2, GRIN3A, SHANK1, RAPGEF4, MYT1L, KCNK12, RAB3B, GOT1, VCPIP1, DLG2, MAGEE1, LEPR, CAMK4, SNRPN, SNCB, LRRC8B, KCNG3, DHRS9, SH2D5, CALN1, NTNG2, SLC17A6, CABP1, YWHAZ, GREM2, ZNF536, SCN2A, C11orf87, CDKN2D, SVIP, IL12RB2, SSTR2, ZRANB1, GFOD2, SOWAHA, LOC375196, MPP7, CABLES1, SUGT1P3, JPH1, RPS6KA5, ANKRD26, CASKIN1, PRKCB, FAXC, CHRNA3, PLCXD3, PPP3R1, UBR3, LOC100288656, TLN2, NXPH2, LARGE1, HECW2, CARMIL2, PWWP3B, ELAVL2, NCOA7, SCAI, FBXL15, RASGEF1C, KCNK1, GLS2, CBFA2T3, ATP2B1, PSD3, REPS2, GRID1, ANO4, SH3BP1, DLGAP3, FAM81A, DOC2A, FSD1L, CPEB1, KCNV1, TUNAR, KIF5A, GALNTL5, RNF150, FKBP1B, KIF5C, RGS6, CPNE6, BEGAIN, MICAL2, SLC6A17, PPP1R16B, LINC01089, RAB14, ACOT4, DOCK9, MATK, CAMK2B, CDH18, HDGFL3, FAAH, PNMA8A, NEUROD6, FAM153B, TAF4B, CRH, PABPC1L2B, FRRS1L, XK, MAP4, FGF12, SLC35F3, ICA1, RYR2, LOC100996385, RTN4RL1, SLC32A1, JPH3, FEZF2, LOC100506851, GNB5, CCDC184, LINC01616, KRT17, SLITRK4, CD200, SYT13, BICDL1, PITPNM3, GABRA2, NOS1, AGTPBP1, SYN2, CFC1, ARHGEF12, TMEM151B, NRIP2, SH3GL3, LOC283045, LIN7B, EPHA5, CDS1, PEG3, CAMSAP1, LYPD8, CUX2, MAP7D2, SEPTIN8, PRMT8, ACTL6B, MID2, FGF7P3, PAK1, HPCAL4, GARNL3, ZNF385B, KRT222, MTUS2, MCTP1, COX5A, PRKAG2-AS1, MAP4K3-DT, KDM5B, CACNA1D, SGSM1, COX11, HABP4, CPEB3, GRM7, RAB37, TEK, BTBD8, DRD1, DOC2B, C5orf64, BHLHE22, HS3ST4, JAKMIP3, SYP, CRYM, HCG11, GPR83, PENK, CDKL5, SDR16C5, STAT4, CHIC1, SRRM4, ABCG4, RAB3C, SPHKAP, USP15, ANKRD13C, ATOH7, RIMS2, SLC25A27, EPB41L4B, ITPKA, GPR22, CCDC85A, PTPN20, NEGR1, KCNJ9, STMN4, AP4S1, SSX2IP, CYFIP2, RTN4R, CNNM2, GNAO1, CACNA1I, ENTPD3, WDR7, GABRB2, SNCA, KIRREL3, PDCD4, ZPR1, ATP2B2, TMEM191A, PACSIN1, SYNPR, C19orf12, DHX30, TMOD2, NYAP1, CELF5, SEPTIN11, RGS8, LINC00957, RAB26, TSPYL1, ANKRD34A, NAPB, FABP6, ANKH, COPS8, STRC, NRG3, HS6ST3, SH3BGRL2, BCL11A, C1QTNF4, CACNA2D3, TAGLN3, CNTN6, CACNA1C, PPP2R2C, TPM3, SHANK2, SCN8A, BCL11B, NRXN3, NAP1L5, SPIRE2, NELL1, SERP2, LINC01128, AAK1, SLC26A4-AS1, RIIAD1, KCNJ18, SORBS2, KLF8, CLSTN3, UBE2QL1, ATP2B3, CNTN4, PCLO, SHISAL1, B4GALNT1, AKAP6, SCN2B, RUNX1T1, TRPM3, CLUHP3, OPCML, NPTX1, RYBP, HTR2A, DLGAP1, ZFP90, MDH1, HTR1E, ADCY1, CLEC2L, CA10, PPP5D1P, MCF2L, CCDC149, CALB1, SEC61A2, ESYT3, ULK2, KIAA0513, CES4A, ENTREP2, SLC24A2, ATP6V1A, CYRIB, BCLAF1, MEG3, PPFIA2, FBXW7, SV2C, CDKL1, PRR36, CYP4X1, KCTD16, VWA5B2, THEMIS, NEFH, SRCIN1, RGS7BP, MACROD2, SLC7A10, ARRB1, MAPK10, TSPOAP1, ATPAF1, MEAF6, LOC101929748, KLHL1, CNNM1, SYCE1, CPLX3, SYT4, TINCR, MFSD4A-AS1, PRKACB, PAK5, SGPP2, GABRG1, KLC1, DLG4, TESPA1, CLASP2, KIAA1549L, STX1A, FABP3, ZFYVE9, GARIN5A, KCNS2, OLA1, CKMT1B, TMEFF2, SPTB, CNKSR2, GRIN1, KCNK3, GRK3, CADPS, SYNE1, SYT3, LRRC7, KCNIP3, CRHBP, SVOP, PHF24, GABRB3, NR4A2, GRIN2A, TLCD3B, UBE3A, MAPK9, GNAL, KSR2, DTNB, LOC103344931, SLC25A4, LINC01260, HMGCLL1, ANAPC1, CELF4, PPEF1, KCNAB1, PIGY-DT, PNMA8B, DNAJA4, CCNG2, STXBP5L, RBFOX1, PRPH2, ARHGEF7, TPPP, SSBP2, TPTE2P5, MED14, NIPSNAP3B, TMEM37, SLC17A7, ANXA11, LINC00294, YJEFN3, FAF1, TSC22D1-AS1, FGF9, ATP6V1G2, KRTAP5-AS1, KCNJ6, MARCHF11, LHX6, SLIT3, MAST1, SEMA3G, PPP1R1A, CDC42, LNX1, ZCCHC12, HCN1, EPCAM, NAV3, NALF1, EFNA5, AIFM3, PDE1A, CUL3, FRMPD4, ATAD1, CRIM1-DT, ZNF280B, NSF, CLCN4, ECPAS, HOOK1, MCF2, NHSL2, PPP4R4, ZNF519, AFF2, FGF14, ARL6, PAK6, LMO7, SLC6A15, WNK2, MAGI1, GPR180, NUDT18, NUP93, PTPN4, GABRA4, UNC79, WSCD2, TLCD4, ATP8A2, CNTNAP2, RBFOX3, MICOS10, HS3ST5, PVR, ZDHHC8BP, MCHR2, CYB5B, TRHDE-AS1, CACNG2, GRM1, CYP2E1, GFOD1, SNAP91, INPP5F, CYS1, ARHGAP44, CAMK2A, RAB11FIP4, PPP3CA, GABRD, SLC45A4, NEUROD2, CACNA1G, BSPRY, BSN, PRSS3P2, TPD52, LOC100507547, KLHL35, PRSS3P1, POLR3A, FBXO41, HSPA12A, SLC29A3, HTR5A, VSNL1, SLC1A6, HAR1A, RALYL, UNC5A, ATF7IP2, ZNF589, FGF13, AGMAT, BEX2, FBXO27, KCNA1, PTGER3, TTC9B, SGIP1, KCNMA1, SIRT5, RIMS1, LOC101926887, LINC02693, JAKMIP1, DGKE, PAH, TTC9, LRTM2, PHACTR1, LOC105374336, N4BP2L1, PRKCE, CHRNA7, LMO3, RXFP1, CLEC4G, PKP4, ADCYAP1, KIF3A, UNC13A, PPM1J, BEX5, DNAJC6, FXR1, RAP1GAP2, MFSD6, SYT9, SNHG28, GPR85, MKX, LAS1L, AGBL5, ATRNL1, EPB41L1, LOC283683, PDE1B, IPCEF1, PARVB, MAL2, RELN, GAD2, FSTL5, FNDC9, SMIM8, OLFM3, STXBP5, DOK6, OGDHL, LINGO2, MAP3K21, FBXO45, PGBD5, PRKAR1B, LINC00515, IDS, ATXN3, PIN1P1, MADD, DGCR5, STK3, PPP2R2D, EIF4E3, CYP26B1, BRAF, TPTE2P1, GRM2, NWD2, AGAP2, CDKL2, RPA1, CARTPT, TMEM132D, CDK5R1, BORCS8, EGR4, MAPK8IP2, RAB6B, SAMD12, WAC, PPP1R13B, SYNGR3, CBLN1, IQSEC2, KCNJ12, OPRK1, STPG1, ZNF385D, GRIN2C, RUNDC3B, CDH12, CPNE9, DSTN, ARMCX5, ACTR3B, STYK1, SRD5A1, SGTB, SLC2A12, FBXL14, CINP, NEFL, GALNT9, NT5DC1, ISLR2, SV2B, PTPRR, RHOBTB2, MIR7-3HG, CAMSAP3, GUCY1B1, YLPM1, PVALB, CDH8, KCNQ2, MMP24, RPS6KA6, SMIM10L2A, FOXQ1, CCKBR, TTC5, ARPP21, ATP6V1D, FRMPD2, CRTAC1, XKR4, KCNK9, NGB, SHISA8, PTPRD, HTR2C, KHDRBS2, DMRTC1, LANCL1, GNAZ, TSPOAP1-AS1, CAMKK1, SCG5, PCNX4, NPM2, NRIP3, GSTO2, ADARB2, ARPP19, AMER3, SYT1, SCN3B, SEZ6L2, HECW1, EPHA7, OIP5-AS1, ATL1, GABRG2, FAM174B, RASL11B, RPL15, CDH22, RSPO3, GDF10, LINC01106, SLC25A42, LONRF2, VWC2, CRYZL2P, VIP, MRAP2, GLT1D1, ATP6V1E1, RNF115, RGS7, LPCAT4, HAPLN4, TRHDE, ADAM11, PCP4L1, RASGRP1, RAB11FIP1, CABYR, OTUD7A, CCDC177, SYNGR1, SLC8A2, CAMKK2, GHITM, PTPN3, STXBP6, LRRC20, CDH9, CACNB4, USP51, CACNA1B, ZNF540, SVIL-AS1, NUAK1, TCERG1L, DLG3, FILIP1, RIMS3, CAP2, SNCG, GNL1, SLITRK1, MBD5, ZC3H12B, KCNIP4, BRSK2, EIF4E, LOC100506563, CNTNAP4, MAP3K9, C12orf76, CYGB, CNTNAP5, LINC00507, RFPL3S, ANK1, RBM11, RAB40B, KCNB2, RIMKLA, UNC5C-AS1, DCTN1-AS1 |

**Table S4.** List of DEGs by WGCNA.

| SOX2, NUSAP1, CDCA3, PIK3AP1, KNTC1, STK36, DDAH2, SYNRG, IL13RA1, SP100, PDE1A, CMTM3, RIN3, MAN2B1, MYO1C, MCM8, SMIM3, PHF19, CHST14, DRAM1, VAMP5, PCLAF, ACOT9, GIMAP2, HLADQ-B1, PSKH1, CD300A, CNPY4, GPR173, CYP20A1, NAGA, UBQLN4, PSMB8, PSMB9, FXYD5, NBN, CLDN15, TRAC, CHEK1, STING1, ZNF227, FBXO17, MCM10, SST, RREB1, PCGF3, NEDD1, KCTD11, PARVG, LRCH1, CHRM1, HNMT, TRIP10, LILRB1, NCAPG2, IKBIP, RYR2, ZNF551, HMG20B, NRP2, STAB1, FARP2, DDX11, CACNG3, CD84, PRKD3, DNAAF5, CKMT1B, HPCA, STK10, BMP1, PARP4, TMEM161A, BTN2A2, UBA7, ZNF117, H2BC6, LMF2, PCCA-DT, STXBP2, GLA, NOTCH2NLB, METRN, FBXO4, ACTMAP, FCGR2A, NFATC3, AFG3L1P , ECSCR, CLN3, ALDH16A1, RAD54B, MKLN1, SYK, TSPO, PXN, MAP3K9, SDF2L1, NID1, AK2, PYGL, TIMELESS, LRRC7, CCDC97, APOBEC3C, C1QTNF5, CTC-338M12.4, ALDH3B1, BSN, DMWD, MMP14, RAB32, MS4A6A, TAF1L, MVP, UAP1L1, HOMER3, N-acetyl, TARBP2, ILVBL, SLC39A7, VAV2, HSD17B8, FERMT3, CHST3, PMM2, EIF4E2, CDCA4, COL4A1, FKBP10, NEMP1, Hsp40, FLNA, TCF3, MIR34AHG, MIR4453HG, RBFOX3, HTR2A, SPOUT1, BCL2L11, RNASEH2A, KDM5A, TROAP, POLM, NSD2, CAMK2A, ANXA2P2, CNOT10, DTYMK, SH3BP2, TMEM51, RHOBTB3, FPGS, PKP4, CAPG, GTSE1, UBE2C, EIF1AD, GMFG, TMEM175, NUP98, CASP1, NMD, CENPU, OBSL1, ZNF300, CASP6, LAMB2, LAMC1, SIVA1, CCDC80, OPA3, CHST11, NUP214, LMNB2, SLC43A3, CORO1B, SPECC1L, LCAT, TEAD4, COLGALT1, LYAR, RAB20, SLC7A7, CPT2, RFNG, ATAD2, CDCA8, PMS2CL, BTN3A2, MXD4, SYDE1, TRIM5, GPATCH2L, PPP4R4, COG8, ZNF3, MICALL1, TGFB1I1, LIG1, RAD51AP1, BORCS8, MYOF, ARHGDIG, LRR1, LIMK2, RNASE2, FAM153B, RNASE6, ARRB1, KLHL25, EVA1B, ANGPTL2, G6PC3, CCNB2, FANCI, LPCAT3, COMMD2, RASSF1, VSTM2L, TRIM56, PRIM1, EIF4A1, CBLN2, TLR2, TLR5, CD44, TSPAN4, SASS6, UHRF1, KIAA0040, SMARCD2 , POLD4, SLC35F2, PCP4L1, LTBP1, TMEM39A, RIMS1, AP4M1, TMEM35B, MAPK7, ESPL1, CMTM7, ATP2B2, SNRNP200, IL4R, PALLD , SNTB1, CDK2, GABRA1, SNX5, B4GALT4, C4orf46, GABRA5, KIDINS220, FOXM1, CCDC71L, GPLD1, MAL2, NAT1, MAPRE3, ASXL1, FGFR1OP2, NIBAN1, CACNB4, FRY, PRMT8, NEUROD1, KLHL1, GLS2, ANK1, ANK3, NDC80, RASGRF1, GFOD1, KLHL7, CLIC1, BAZ1A, SV2B, RBL1, NUP37, KCNJ3, HCN1, ATP8A2, KCNJ9, DTL, SLITRK4, NEURL1, KCNK1, PBK, ASPM, SLC17A7, TMEM266, KIF14, CASP8, KIF4A, MFSD4A, DLG2, CRNDE, RCC1, C9orf24, ACTL6A, CDK1, KIF5C, TNFAIP8, PRR11, KCNJ18, SNCA, PABPC1L2B , RRM2, EZH2, MARCHF11, PRRT1 , MELK, SVOP, DGCR5, PTPN20, SLC1A6, PABIR2, TOP2A, ZNF702P, MAPK8IP2, KIF11, IQGAP1, GABRA4, MAP7, EPHA6, S100A11P1, CATSPER2, MYT1L, GABRB2, GABRG1, GABRG2, GAD2, CDS1, PTTG1, NMNAT2, CELF4, SGSM1, ARPP21, BTBD8, RPS6KA5, GLT1D1, CENPF, SRRM4, LINC00957, FSTL5, GJB6, MBP, HLF, CPLX2, ANKS1B, SYT9, PHYHIP, SAMD12, DDN, PAIP2B, SSTR1, NKAIN2, PTER, CYP46A1, PRR18, CCDC85A, NCDN, SLC12A5, KIAA0319, AURKA, CDKL5, UNC5A, GPR83, DUSP26, CKS1B, CKS2, NEK2, RUNDC3A, NCAPG, KCNA1, AJAP1, NALCN, NECAB1, SLC24A2, ARHGEF7, SPARC , LRFN5, CLVS2, AK5, SCN2B, BHLHE22, ARHGAP44, KRT222, ELAPOR1, OPALIN, PCDH11X, KCNMA1, FBXO41, SYN1, SYN2, MAP7D2, KCNQ3, SGO2, CREG2, KIF5A, GNAL, HS3ST4, SULT4A1, RAB3A, MOBP, HPCAL4, CALN1, PGBD5, NPY, MPPED1, PARM1, CNDP1, SHANK1, KDELR2, CACNA1B, SLC25A27, CACNA1E, LIMA1, STUM, GPR26, KALRN, WEE1, PACSIN1, STXBP5, SMIM10L2A, PHF24, PCLO, SH3GL3, CAPN3, VSTM2A, AAK1, CPEB3, KCNAB2, SMC4, MYRIP, RASGEF1A, SLC6A15, DGKE, XK, SRCIN1, LINGO2, GRIN2A, GRIN2B, BCAS1, PRRT2, GRM5, PEX5L, ECT2, ST8SIA3, SLC7A14, RGS7, CCK, CCKBR, SPX, CCNA2, CCNB1, PPP1R1A, PDIA4, IL1RAPL1, PPP2R2C, BCL11B, GALNT17, LMO7, CADPS, IPCEF1, TTLL11, RBFOX1, WIF1, OTUD7A, CNTNAP2, CNTNAP4, RACGAP1, PRKCB, RIMBP2, UNC13C, NUP93, RAB11FIP4, NRSN1, NRG3, ATP1A3, REPS2, ABLIM2, EMX2, CDC42, HS6ST3, GOLGA8A, FAIM2, LSM8, ANO3, RFPL1S, MAD2L1, PCNA, SCN3B, CDH18, JPH3, CPEB1, GABRA2, BORA, CDK6, TMEM88B |
| --- |

**Table S5.** List of common DEGs (cDEGs) between two approaches (ML and WGCNA).

| DTL, CENPF, BMP1, ASPM, CDK1, IQGAP1, ASXL1, SMC4, RNASEH2A, MCM8, TCF3, LTBP1, PCLAF, ALDH3B1, NCAPG, TNFAIP8, CMTM3, NAGA, DDX11, CCNB2, CDK6, NID1, ANGPTL2, NRP2, SP100, EIF4A1, SYDE1, RRM2, CCDC80, MCM10, NDC80, GTSE1, CHEK1, WEE1, CHST14, FARP2, PRKD3, CNPY4, IKBIP, LAMC1, EIF4E2, NIBAN1, KIAA0040, TEAD4, NEMP1, TOP2A, PMM2, BORA, STING1, TGFB1I1, CD44, CENPU, LRR1, TRIM5, DRAM1, IL13RA1, SLC35F2, PBK, NEDD1, CDCA8, PSMB8, AURKA, SLC7A7, CDCA3, NUSAP1, FBXO4, RAD51AP1, ACTMAP, MICALL1, KIF14, ESPL1, NSD2, MYOF, NAT1, SPOUT1, TRIP10, MAPK7, UBE2C, TIMELESS, PABIR2, CRNDE, CHST11, FANCI, ANXA2P2, TROAP, KNTC1, RBL1, RCC1, FXYD5, CASP6, MFSD4A, SULT4A1, CACNA1E, GPR26, KCNJ3, ANK3, UNC13C, GABRA1, AJAP1, SHANK1, MYT1L, DLG2, CALN1, RPS6KA5, PRKCB, KCNK1, GLS2, REPS2, CPEB1, KIF5A, KIF5C, CDH18, FAM153B, XK, RYR2, JPH3, SLITRK4, GABRA2, SYN2, SH3GL3, CDS1, MAP7D2, PRMT8, HPCAL4, KRT222, SGSM1, CPEB3, BTBD8, BHLHE22, HS3ST4, GPR83, CDKL5, SRRM4, SLC25A27, CCDC85A, PTPN20, KCNJ9, GABRB2, SNCA, ATP2B2, PACSIN1, LINC00957, NRG3, HS6ST3, PPP2R2C, BCL11B, AAK1, KCNJ18, PCLO, SCN2B, HTR2A, SLC24A2, SRCIN1, ARRB1, KLHL1, GABRG1, CKMT1B, CADPS, LRRC7, SVOP, PHF24, GRIN2A, GNAL, CELF4, RBFOX1, ARHGEF7, SLC17A7, MARCHF11, PPP1R1A, CDC42, HCN1, PDE1A, PPP4R4, LMO7, SLC6A15, NUP93, GABRA4, ATP8A2, CNTNAP2, RBFOX3, GFOD1, ARHGAP44, CAMK2A, RAB11FIP4, BSN, FBXO41, SLC1A6, UNC5A, KCNA1, KCNMA1, RIMS1, DGKE, PKP4, SYT9, IPCEF1, MAL2, GAD2, FSTL5, STXBP5, LINGO2, PGBD5, DGCR5, BORCS8, MAPK8IP2, SAMD12, SV2B, SMIM10L2A, CCKBR, ARPP21, SCN3B, GABRG2, GLT1D1, RGS7, PCP4L1, OTUD7A, CACNB4, CACNA1B, CNTNAP4, MAP3K9, ANK1 |
| --- |

**Table S6**. List of key genes (KGs) from PPI network based on different topological measures

| **SN.** | **Gene** | **Closeness** | **Degree** | **EPC** | **MCC** | **MNC** | **DMNC** |
| --- | --- | --- | --- | --- | --- | --- | --- |
| 1 | ASPM | 87.25 | 37 | 93.565 | 2.36E+21 | 32 | 1.068947 |
| 2 | CCNB2 | 87.11 | 38 | 93.565 | 2.36E+21 | 37 | 0.910688 |
| 3 | CDK1 | 83.12 | 40 | 93.565 | 2.36E+21 | 40 | 0.820328 |
| 4 | AURKA | 79.99 | 37 | 93.565 | 2.36E+21 | 36 | 0.908891 |
| 5 | TOP2A | 78.39 | 34 | 93.474 | 2.36E+21 | 33 | 1.0433 |
| 6 | CHEK1 | 76.40 | 32 | 93.365 | 2.36E+21 | 32 | 1.016466 |
| 7 | CDCA8 | 76.82 | 33 | 93.565 | 2.36E+21 | 33 | 1.051164 |
| 8 | SMC4 | 76.40952 | 32 | 93.565 | 2.36E+21 | 32 | 1.005417 |
| 9 | MCM10 | 76.15952 | 32 | 93.468 | 2.36E+21 | 32 | 1.079995 |
| 10 | RAD51AP1 | 75.54286 | 29 | 93.221 | 1.18E+21 | 28 | 1.154185 |

**Table S7.** Test performance scores of the RF-based prediction model with the cutoff at FPR < 10%

|  | Test Dataset  (40% of metadata samples) | Independent Test Dataset (GSE50161) |
| --- | --- | --- |
| Area under the ROC Curve (AUC) | 0.992 | 0.989 |
| Accuracy (ACC) | 0.93 | 0.92 |
| Sensitivity or true positive rate (TPR) | 0.90 | 0.92 |
| Specificity or true negative rate (TNR) | 0.94 | 0.91 |
| False negative rate (FNR) | 0.04 | 0.07 |
| False positive rate (FPR) | 0.09 | 0.08 |
| False discovery rate (FDR) | 0.09 | 0.08 |

| **Table S8.** The top significantly (p-value<0.05) enriched GO functionals and KEGG pathways by KGs. | | | | | |
| --- | --- | --- | --- | --- | --- |
| **Biological Process** | | | | | |
| GO ID | **Description** | **p-value** | | | **Annotated KGs** |
|  |  | **Gene**  **Codis** | **David** | **Enrichr** |  |
| GO:0000086 | G2/M transition of mitotic cell cycle | 2.69E-06 | 3.12E-04 | 1.57E-04 | CDK1, CHEK1, AURKA |
| GO:0010569 | regulation of double-strand break repair via homologous recombination | 3.76E-05 | 0.008315 | 5.49E-04 | RAD51AP1, CHEK1 |
| GO:0006260 | DNA replication | 4.76E-05 | 0.054726 | 5.80E-04 | CDK1, CHEK1, MCM10 |
| GO:0006281 | DNA repair | 0.001155 | 0.007822 | 3.39E-04 | CDK1, RAD51AP1, CHEK1 |
| GO:1901796 | regulation of signal transduction by p53 class mediator | 0.002711 | 0.018851 | 2.40E-04 | CHEK1, AURKA |
| **Cellular Component** | | | | | |
| GO ID | **Description** | **p-value** | | | **Annotated KGs** |
|  |  | **Gene**  **Codis** | **David** | **Enrichr** |  |
| GO:0005634 | nucleus | 2.88E-05 | 3.56E-04 | 1.14E-05 | SMC4, ASPM, CDK1, RAD51AP1, CDCA8, CHEK1, AURKA, TOP2A, CCNB2, MCM10 |
| GO:0000307 | Cyclin-Dependent Protein Kinase Holoenzyme Complex | 0.000205 | 0.018612 | 1.74E-04 | CDK1, CCNB2 |
| GO:0005876 | spindle microtubule  in apoptotic signaling pathway | 0.000905 | 0.019471 | 5.04E-04 | CDK1, AURKA |
| GO:0000794 | condensed nuclear chromosome | 0.000269 | 0.016894 | 8.45E-05 | SMC4, CHEK1 |
| GO:0015630 | microtubule cytoskeleton | 0.000114 | 0.002973 | 0.001142 | CCNB2, CDCA8, AURKA |
| **Molecular Function** | | | | | |
| GO ID | **Description** | **p-value** | | | **Annotated KGs** |
|  |  | **Gene**  **Codis** | **David** | **Enrichr** |  |
| GO:0003697 | single-stranded DNA binding | 2.45E-05 | 0.001235 | 0.001001 | SMC4, RAD51AP1, MCM10 |
| GO:0004674 | protein serine/threonine kinase activity | 0.001218 | 0.014374 | 0.015009 | CHEK1, CDK1, AURKA |
| GO:0004712 | protein serine/threonine/tyrosine kinase activity | 0.001572 | 0.01739 | 0.012927 | CDK1, CHEK1, AURKA |
| **KEGG Pathways** | | | | | |
| KEGG ID | **description** | **p-value** | | | **Annotated KGs** |
|  |  | **Gene**  **Codis** | **David** | **Enrichr** |  |
| hsa04115 | p53 signaling pathway | 7.74E-06 | 4.47E-04 | 5.50E-06 | CDK1, CHEK1, CCNB2 |
| hsa04110 | Cell cycle | 4.01E-05 | 0.002001 | 7.61E-05 | CDK1, CHEK1, CCNB2 |
| hsa04914 | Progesterone-mediated oocyte maturation | 2.13E-05 | 8.49E-04 | 1.42E-05 | CDK1, AURKA, CCNB2 |
| hsa05203 | Viral carcinogenesis | 0.006268 | 0.092984 | 0.004372 | CHEK1, CDK1 |

| Table S9. Methylation Status of the KGs in GBM by MethSurv. | | | | | |
| --- | --- | --- | --- | --- | --- |
| sKGs | **Gene_Group** | **CpG_Island** | **CPG Name** | **HR** | **P-Value** |
| ASPM | TSS200 | Open_Sea | cg16552589 | 0.661 | 0.04914 |
| CCNB2 | TSS200 | Island | cg17236576 | 0.566 | 0.02139 |
| CDK1 | TSS200 | Island | cg06793798 | 0.599 | 0.01476 |
| CDK1 | TSS200 | Island | cg13227473 | 0.661 | 0.05063 |
| CDK1 | TSS200 | Island | cg13954297 | 0.503 | 0.00725 |
| AURKA | Body | Open_Sea | cg09712306 | 0.556 | 0.01905 |
| TOP2A | Body | Island | cg11393025 | 0.505 | 0.00714 |
| CHEK1 | TSS1500 | Island | cg03474731 | 0.625 | 0.05286 |
| CDCA8 | TSS200 | Island | cg27170383 | 0.538 | 0.01140 |
| MCM10 | TSS1500 | N_Shore | cg12241367 | 0.610 | 0.01858 |
| MCM10 | 5'UTR | S_Shore | cg17109175 | 0.665 | 0.05418 |
| RAD51AP1 | Body | S_Shelf | cg14552441 | 0.565 | 0.02282 |

**Table S10**. Lipinski rule of 5. The drugs highlighted in bold font satisfied all the five rules.

| No | Drug name | mass | hydrogen bond donor | hydrogen bond acceptors | LOGP | Molar Refractivity | violation |
| --- | --- | --- | --- | --- | --- | --- | --- |
| 1 | BHG712 | 503.00 | 2 | 7 | 5.558 | 132.73 | 3 |
| 2 | YM201636 | 467.00 | 3 | 10 | 3.31 | 131.51 | 1 |
| 3 | GSK2126458 | 505.00 | 1 | 8 | 5.92 | 129.76 | 2 |
| 4 | CX5461 | 513.00 | 1 | 8 | 3.43 | 144.20 | 2 |
| 5 | irinotecan | 586.00 | 1 | 10 | 3.70 | 157.98 | 2 |
| 6 | imatinib | 493.00 | 2 | 8 | 4.40 | 146.23 | 1 |
| 7 | **SNX2112** | **464.00** | **4** | **6** | **4.05** | **115.56** | **0** |
| 8 | NG25 | 537.00 | 2 | 5 | 6.07 | 135.35 | 3 |
| 9 | **vatalanib** | **346.00** | **1** | **4** | **4.24** | **98.38** | **0** |
| 10 | GSK1070916 | 507.00 | 2 | 6 | 5.93 | 153.95 | 3 |
| 11 | Midostaurin | 570.00 | 1 | 7 | 5.96 | 164.45 | 3 |
| 12 | Masitinib | 498.00 | 2 | 7 | 5.06 | 146.31 | 1 |
| 13 | **crenolanib** | **443.00** | **7** | **1** | **3.91** | **110.10** | **0** |
| 14 | AZ628 | 451.00 | 2 | 7 | 5.53 | 134.18 | 2 |
| 15 | xl147 | 448.00 | 2 | 8 | 5.56 | 122.26 | 1 |
| 16 | etoposide | 588.00 | 3 | 13 | 1.15 | 137.41 | 3 |
| 17 | cabozanitinib | 501.00 | 2 | 8 | 5.54 | 136.68 | 3 |
| 18 | dabrafenib | 520.00 | 2 | 7 | 5.36 | 130.25 | 3 |
| 19 | **MLN4924** | **443.00** | **4** | **8** | **2.91** | **114.59** | **0** |
| 20 | **TGX221** | **364.00** | **1** | **5** | **2.44** | **103.98** | **0** |
| 21 | **fenbendazole** | **299.00** | **2** | **4** | **3.89** | **82.68** | **0** |
| 22 | **RO3306** | **351.00** | **1** | **4** | **4.05** | **100.91** | **0** |
| 23 | UNC0638 | 509.00 | 1 | 7 | 5.83 | 151.05 | 3 |
| 24 | **fluoxetine** | **309.00** | **1** | **2** | **4.43** | **79.79** | **0** |
| 25 | **vandetanib** | **474.00** | **1** | **6** | **5.00** | **119.18** | **0** |

| **Table S11**. The top ranked receptor protein (AURKA) and top 4 lead compounds (Fluoxetine, Vatalanib, TGX221 and RO3306) based on ADMET and docking results. The third column displays the 3D structure of receptor protein AURKA along with potential therapeutics. The neighboring residues (within 4 Å of the drug) are displayed in the fourth column by the 2D Schematic representation of the receptor-drug interactions. The final column displayed the interaction types and amino acids of the receptor proteins which took part in those interactions. | | | | | | | |
| --- | --- | --- | --- | --- | --- | --- | --- |
| Drug-target complexes | Binding  Affinity (Kcal/  mol) | Complexes three dimentional  (3D) view. | 2D view of the receptor-Drug interactions | Interaction types and interacting amino acids. | | | |
|  |  |  |  | Hydro-gen  bond | Hydro-  phobic  inter-action | Halogen/  Salt Bridge | Π-stack-ing |
|  |  |  | 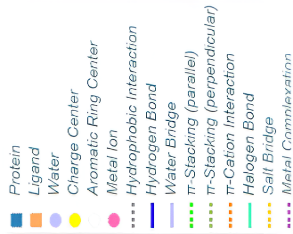 |  |  |  |  |
| AURKA- RO3306 | -8.90 | 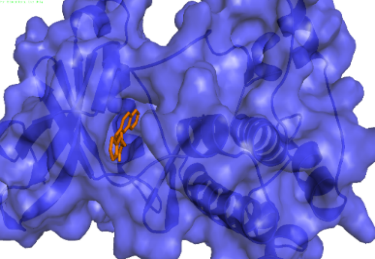 | 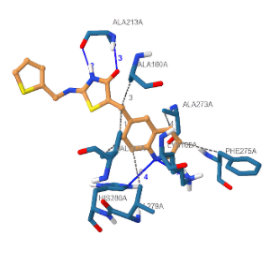 | LYS162  ALA213  HIS280 | VAL147  ALA160  ALA273  PHE275  VAL279 | - | - |
| AURKA-Vatalanib | -9.70 | 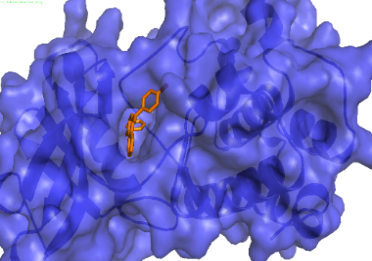 | 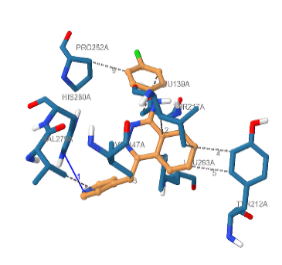 | HIS280 | LEU139  VAL147  TYR212  THR217  LEU263  VAL279  PRO282 | - | - |
| AURKA-TGX221 | -8.80 | 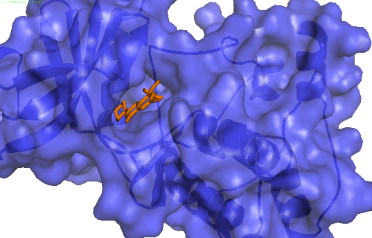 | 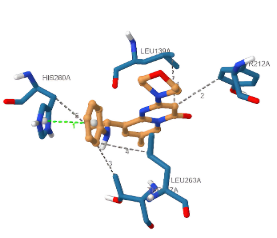 | - | LEU149  TYR212  THR217  LEU263  HIS280 | - | HIS280 |
| AURKA-Fluoxetine | -8.20 | 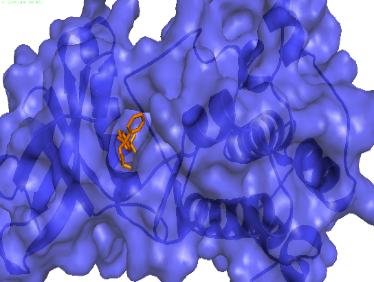 | 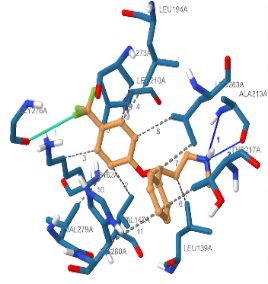 | ALA213 | LEU139  VAL147  LYS162  LEU194  LEU210  THR217  LEU263  ALA273  VAL279  HIS280 | GLY276 |  |

**Reference:**

1. Staedtke, V., Bai, R. Y. & Laterra, J. Investigational new drugs for brain cancer. *Expert Opin. Investig. Drugs* **25**, 937–956 (2016).

2. Juillerat-Jeanneret, L. The targeted delivery of cancer drugs across the blood-brain barrier: chemical modifications of drugs or drug-nanoparticles? *Drug Discov. Today* **13**, 1099–1106 (2008).

3. Zhuo, C. *et al.* Surprising anticancer activities of psychiatric medications: Old drugs offer new hope for patients with brain cancer. *Front. Pharmacol.* **10**, 1–7 (2019).

4. Kim, C., Shah, B. P., Subramaniam, P. & Lee, K. B. Synergistic induction of apoptosis in brain cancer cells by targeted codelivery of siRNA and anticancer drugs. *Mol. Pharm.* **8**, 1955–1961 (2011).

5. Wainwright, D. A., Nigam, P., Thaci, B., Dey, M. & Lesniak, M. S. Recent developments on immunotherapy for brain cancer. *Expert Opin. Emerg. Drugs* **17**, 181–202 (2012).

6. Sontheimer, H. & Bridges, R. J. Sulfasalazine for brain cancer fits. *Expert Opin. Investig. Drugs* **21**, 575–578 (2012).

7. Newton, H. B. Novel chemotherapeutic agents for the treatment of brain cancer. *Expert Opin. Investig. Drugs* **9**, 2815–2829 (2000).

8. Laquintana, V. *et al.* New strategies to deliver anticancer drugs to brain tumors. *Expert Opin. Drug Deliv.* **6**, 1017–1032 (2009).

9. Benjamin, W. No Titיליle. ペインクリニック学会治療指針２ **3**, 1–9 (2019).

10. Fung, N. H. *et al.* Understanding and exploiting cell signalling convergence nodes and pathway cross-talk in malignant brain cancer. *Cell. Signal.* **57**, 2–9 (2019).

11. Bahmad, H. F. *et al.* Repurposing of Anticancer Stem Cell Drugs in Brain Tumors. *J. Histochem. Cytochem.* **69**, 749–773 (2021).

12. Yoo, M. *et al.* DSigDB: Drug signatures database for gene set analysis. *Bioinformatics* **31**, 3069–3071 (2015).

13. Liu, C. J. *et al.* GSCALite: A web server for gene set cancer analysis. *Bioinformatics* **34**, 3771–3772 (2018).
